# Sialic Acid-Siglec-E Interactions During *Pseudomonas aeruginosa* Infection of Macrophages Interferes With Phagosome Maturation by Altering Intracellular Calcium Concentrations

**DOI:** 10.3389/fimmu.2020.00332

**Published:** 2020-02-28

**Authors:** Kaustuv Mukherjee, Biswajit Khatua, Chitra Mandal

**Affiliations:** Cancer Biology and Inflammatory Disorder Division, CSIR-Indian Institute of Chemical Biology, Kolkata, India

**Keywords:** *Pseudomonas aeruginosa*, sialic acids, sialic acid-binding immunoglobulin-like lectins (siglecs), siglec-E, macrophage, calcium, phagosome-lysosome fusion, signaling pathways

## Abstract

*Pseudomonas aeruginosa* (PA) is commonly associated with nosocomial and chronic infections of lungs. We have earlier demonstrated that an acidic sugar, sialic acid, is present in PA which is recognized and bound by sialic acid binding immunoglobulin type lectins (siglecs) expressed on neutrophils. Here, we have tried to gain a detailed insight into the immunosuppressive role of sialic acid-siglec interactions in macrophage-mediated clearance of sialylated PA (PA^+Sia^). We have demonstrated that PA^+Sia^ shows enhanced binding (~1.5-fold) to macrophages due to additional interactions between sialic acids and siglec-E and exhibited more phagocytosis. However, internalization of PA^+Sia^ is associated with a reduction in respiratory burst and increase in anti-inflammatory cytokines secretion which is reversed upon desialylation of the bacteria. Phagocytosis of PA^+Sia^ is also associated with reduced intracellular calcium ion concentrations and altered calcium-dependent signaling which negatively affects phagosome maturation. Consequently, although more PA^+Sia^ was localized in early phagosomes (Rab5 compartment), only fewer bacteria reach into the late phagosomal compartment (Rab7). Possibly, this leads to reduced phagosome lysosome fusion where reduced numbers of PA^+Sia^ are trafficked into lysosomes, compared to PA^−Sia^. Thus, internalized PA^+Sia^ remain viable and replicates intracellularly in macrophages. We have also demonstrated that such siglec-E-sialic acid interaction recruited SHP-1/SHP-2 phosphatases which modulate MAPK and NF-κB signaling pathways. Disrupting sialic acid-siglec-E interaction by silencing siglec-E in macrophages results in improved bactericidal response against PA^+Sia^ characterized by robust respiratory burst, enhanced intracellular calcium levels and nuclear translocation of p65 component of NF-κB complex leading to increased pro-inflammatory cytokine secretion. Taken together, we have identified that sialic acid-siglec-E interactions is another pathway utilized by PA in order to suppress macrophage antimicrobial responses and inhibit phagosome maturation, thereby persisting as an intracellular pathogen in macrophages.

## Introduction

Sialic acid is a nine-carbon acidic sugar usually found at the terminal positions of carbohydrate chains which decorate many cell surface and secretory proteins of higher vertebrates. However, certain pathogenic bacteria such as *Campylobacter jejuni*, group B Streptococci and *Neisseria* synthesize sialic acids and utilize them to interact with sialic acid-binding immunoglobulin-like lectins (siglecs) on host cells leading to immune suppression for successful establishment of infection (1–4). Other bacteria like *Haemophilus influenzae* are known to acquire sialic acids from their environment (5). We have previously demonstrated the presence of sialic acids on *Pseudomonas aeruginosa* (PA), a ubiquitous Gram-negative bacterium (6). These sialylated PA (PA^+Sia^) interact with siglecs on neutrophils and reduce complement deposition (7). Such binding also impairs NETs formation, cytokine secretion, ROS generation as well as other biological functions thereby aiding their survival (8).

In general, macrophages respond to invading bacteria by secreting various cytokines, chemokines and attempt to eliminate such bacteria through phagocytosis followed by generation of reactive oxygen and nitrogen species (9, 10). Such phagocytosed bacteria are also trafficked into the endocytic pathway where acidification of phagosomal lumen, addition of degradative enzymes and fusion with the lysosome result in killing of most kinds of bacteria.

PA commonly infects immunocompromised and burn patients (11, 12). They are usually found in lungs of cystic fibrosis patients as chronic infections (13). Alveolar macrophages play a critical role in managing such infections (14). PA produces secondary metabolites and several virulence factors which modify host cell responses during infection. In this context, it is worthwhile to investigate if sialic acid in PA^+Sia^ can modulate macrophage immune responses against it by exploiting siglecs present on macrophages.

Here, we demonstrate that PA^+Sia^ shows enhanced binding to macrophages through sialic acid-siglec-E interactions. Involvement of such sialic acid-siglec-E results in suppression of macrophage respiratory burst and reduced pro-inflammatory cytokines secretion. Additionally, this interaction resulted in the reduction of intracellular calcium ion concentrations during phagocytosis, modulating calcium-based cellular signaling thereby preventing the process of phagosome-lysosome fusion. Moreover, these sialic acid-siglec interactions recruited SHP-1/SHP-2 phosphatases at the immunoreceptor tyrosine-based inhibitory motifs (ITIM) of siglec-E. These phosphatases then interfere with MAPK, ERK, JNK-SAPK, and NF-κB pathways in PA^+Sia^-infected macrophages. Many of these observations were rescued by interrupting sialic acid-siglec-E interaction through silencing siglec-E. Taken together, we have established sialic acid as one of the important molecules utilized by PA to escape host innate immune response leading to its intracellular persistence in macrophages.

## Materials and Methods

### Reagents

Fluorescein isothiocyanate (FITC), Fluo-3-AM, Fura-2-AM, pluronic F, bovine serum albumin (BSA), 4′,6-diamidino-2-phenylindole (DAPI), trypan blue, paraformaldehyde, ionomycin, and cytochalasin-D were obtained from Sigma (St. Louis, MO). *Arthrobacter ureafaciens* sialidase was from Roche Applied Science (Mannheim, Germany); Mounting medium was from Amersham Biosciences (Uppsala, Sweden); 2′7′- dichlorodihydro fluorescein diacetate chloromethyl ester (CM-H_2_DCFDA), 4-Amino-5-Methylamino-2′,7′-Difluorofluorescein Diacetate (DAF-FM Diacetate), Lysotracker Red DND-99, carboxyfluorescein succinimidyl ester (CFSE) dye, Alexafluor-647 conjugated anti-Rabbit secondary antibody was from Molecular Probes, Thermo Fisher Scientific (OR, USA); siglec-E siRNA and anti-siglec-E antibody was from Santacruz Biotechnology (Texas, USA). All cytokine ELISA kits, CD16/32, CD11b, SHP-2, and SHP-1 antibodies were obtained from BD pharmingen and BD Biosciences (San Jose, CA, USA). Anti-Siglec antibodies (siglec-1, 3, 5, 7, 9, and siglec-1 and E) were from R&D systems (MN, USA). All other antibodies were from Cell Signaling Technologies (MA, USA) unless indicated otherwise. All cell culture medium, fetal calf serum (FCS), lipofectamine, and other reagents were purchased from Invitrogen (Thermo Fisher Scientific, Waltham, MA, USA). West-pico enhanced chemiluminescent substrate (ECL) and BCA assay kit was purchased from Pierce (Thermo Scientific, Waltham, MA, USA).

### Bacterial Culture

*Pseudomonas aeruginosa* strain PA14 is a virulent burn-wound isolate, gifted by Prof. Richard D. Cummings from the Emory University School of Medicine (Atlanta, GA, USA). Bacteria were overnight cultured in tryptic soy broth in shaking condition at 37°C to obtain non-sialylated PA (PA^−Sia^).

Additionally, bacteria cultured in tryptic soy broth supplemented with 10% heat-inactivated FCS (Gibco, Thermo Fisher Scientific) were designated as sialylated PA (PA^+Sia^). For infection, overnight grown bacteria were pelleted, washed with phosphate buffered saline (PBS, 0.01 M) and bacterial counts estimated by measuring optical density (OD_600nm_) by spectrophotometry.

### Cell Culture

Murine macrophage (J774A.1) and human monocytic (THP-1) cell lines were obtained from Cell Repository of National Centre for Cell Science, Pune, India and was routinely cultured at 37°C with 5% CO_2_ in IMDM (Gibco, Invitrogen) and RPMI (Sigma) mediums, respectively, supplemented with 10% FCS (15). THP-1 derived macrophages were generated by treating the cells with PMA (100 ng/mL) for 48 h and differentiation was monitored by cellular morphology as well as expression of macrophage-specific cellular markers.

## Estimation of Sialylation in PA

### Lectin Binding Assay

In brief, overnight cultured PA^−Sia^/PA^+Sia^ (1 × 10^7^) were washed, enumerated and then incubated with FITC labeled *Sambucus nigra* (SNA) lectin or *Maackia amurensis* II (MAA) lectin (0.02 μg, Vectorlabs, California, USA) at 4°C for 30 min. Additionally, PA^+Sia^ (1 × 10^7^/100 μL) was desialylated by incubating them with *A. ureafaciens* sialidase (10 mU/100 μL, 10269611001, Roche) for 30 min at 37°C (7) and then used in FITC tagged lectin binding assay. Bacteria were then extensively washed to remove non-specifically bound lectins and analyzed by flow cytometry.

### Acetyl Acetone Based Fluorimetric Estimation

As before, equal numbers of bacteria (5 × 10^9^) were suspended in PBS and lysed by sonication in ice. Protein estimation was done and lysates (500 μg) were used to detect content of sialic acid by the method described by Shukla and Schauer (16). Mild oxidation of lysates by treatment with periodate solution (2.5 mM) at 4°C in 15 min dark was followed by treatment with sodium arsenite (2% in 0.5 M HCl) and acetyl acetone (750 ml glacial acetic acid, 3.75 g ammonium acetate and 500 μl of acetyl acetone in 250 mL water) at 60°C for 10 min then addition of 2.5 mL of water. Sialic acids present in lysates form a fluorogenic product after incubation and this product was quantified using a fluorimeter with excitation at 410 nm and collecting emission at a wavelength of 510 nm.

### FITC-Labeling of Bacteria

Overnight cultured PA^−Sia^ or PA^+Sia^ was labeled with FITC as stated previously (7). Bacteria were pelleted, washed with PBS and its OD_600_ value was determined. Equal numbers of PA^−Sia^ or PA^+Sia^ (1 × 10^9^) were then incubated with 0.1% FITC in a minimal volume of bicarbonate buffer (pH 8.0) for 45 min at 37°C. Unbound FITC was removed by repeated washing with PBS and extent of labeling with FITC was verified by flow cytometry.

Additionally, FITC-labeled bacteria were incubated for 0, 2, or 4 h in PBS at 37°C followed by fixation with 2% para-formaldehyde to preserve FITC signal. Bacteria were analyzed by flow cytometry to detect stability of FITC tag.

### Adhesion and Internalization of FITC-PA^–Sia^/PA^+Sia^ With Macrophages by Confocal Microscopy

J774A.1 cells (5 × 10^4^) were seeded in glass coverslips and incubated overnight in IMDM medium at 37°C with 5% CO_2_. FITC-PA was added to the coverslips at a ratio of 10:1 and incubated at 4°C for 30 min. Unbound bacteria were removed by washing with PBS. These infected macrophages were finally fixed using 2% paraformaldehyde and mounted onto slides using DAPI containing mounting medium to visualize the binding of PA with macrophages by confocal microscopy. All images were acquired by Andor Spinning Disc Confocal microscope (Belfast, U.K.) with a 60 × /1.42 NA oil immersion objective.

In order to check the internalization of bacteria by macrophages, the above experiment was performed by incubating for 30 min at 37°C followed by fixation and staining. Macrophages were also briefly treated with trypan blue solution (0.02%) to quench fluorescence coming from extracellularly adherent bacteria (17). Images were analyzed and bacterial fluorescence associated with individual cells was quantified by Fiji software (18). We have also calculated phagocytic index, which is the mean number of bacteria adhered or internalized per phagocytosing macrophage.

### Phagosome-Lysosome Fusion in Infected Macrophages by Confocal Microscopy

Macrophages were similarly incubated with FITC-PA^−Sia^ or PA^+Sia^ at 10 multiplicity of infection (MOI) and incubated for 15 min at 4°C to synchronize bacterial binding. Cells were then incubated at 37°C for 30 min followed by removal of unbound bacteria by PBS wash. Infected macrophages were further incubated for 60 min. Lysotracker Red DND-99 (100 nM, L7528, Molecular Probes, Invitrogen) was then added and incubation continued for another 30 min at 37°C to label lysosomes. Cells were fixed, mounted on slides, and analyzed by confocal microscopy as mentioned before.

### Detection of Bacterial Localization in Endocytic Pathway by Confocal Microscopy

As previously described, coverslip-adherent macrophages were incubated with 1:10 MOI of FITC-PA^−Sia^ or PA^+Sia^ for 15 min at 4°C. Plates were transferred to 37°C for 10 min to allow synchronous uptake of bacteria and incubated further for 20 min, 40 and 60 min. Cells were fixed, permeabilized using PBS containing 0.3% Triton-X. Cells infected for 20 min were then overnight incubated at 4°C with primary antibodies against Rab5 (1:100, #12666, Cell Signaling Technology) in blocking buffer (PBS containing 10% goat serum and 0.3% Triton-X). Cells infected with PA for 40 min were incubated with anti-Rab7 antibodies (1:100, #12666, Cell Signaling Technology) and those infected for 60 min were incubated with anti-LAMP1 antibodies (1:100, #3243, Cell Signaling Technology). Coverslips were washed with PBS containing 0.3% Triton-X and then counterstained with Alexa Fluor-647 tagged secondary antibodies (1:2000, A-21245, Invitrogen) in blocking buffer for 2 h at 4°C. Coverslips were once again washed, mount and sealed. Images were randomly acquired from multiple fields in Fluoview FV10i (Olympus Life science). In order to confirm co-localization, multiple z stacks of 1 μm distance were also acquired. Images were processed and co-localization analyzed using JaCOP plugin (19) from Fiji software by calculating both Pearson's correlation coefficient (R) and Mander's overlap coefficient. For publication, images were brightened and background subtracted. On an average 100 cells from different fields were analyzed to determine colocalization.

### Intracellular Calcium Levels in Infected Macrophages by Confocal Microscopy

Macrophages were also stained with Fluo-3-AM (5.0 μM, Sigma-Aldrich) for 30 min at 37°C to allow the dye to permeate. They were then infected with PA^−Sia^ or PA^+Sia^ at 1:10 ratio, incubated for 15 min at 37°C before fixation and processing for microscopy. Images were acquired in Fluoview FV10i (Olympus Life science). A similar experimental set up was repeated using siglec-E silenced macrophages for infection with PA^+Sia^. Images were analyzed as discussed above.

## Flow Cytometric Analysis of Phagocytosis

### Binding of PA by Macrophages

J774A.1 (1 × 10^6^) cells were incubated with 10 MOI of FITC-PA^−Sia^ or PA^+Sia^ at 4°C for 30 min to detect their binding. Additionally, PA^+Sia^ (1 × 10^6^) were desialylated as mentioned earlier and used for binding.

To prevent sialic acid-siglec binding, macrophages (1 × 10^6^) were additionally incubated with anti-murine siglec-1 and siglec-E antibodies (1:1,000, R&D systems) for 1 h at 4°C. In the case of THP-1 derived macrophages, anti-Siglec-1, 3, 5, 7, and 9 antibodies (1:1,000, R&D systems) were used. Macrophages were also treated with blocking antibodies against CD11b (complement receptor-3, 550282, BD Biosciences) or CD16/32 (FcγR III/II, 553142, BD Biosciences). Bacteria and macrophages were then allowed to bind at 4°C for 30 min and assessed by flow cytometry in FACS LSR Fortessa (BD Biosciences) and analyzed using FACSDiva 8.0.2 software.

### Internalization of PA by Macrophages

Macrophages were incubated with 10 MOI of FITC-PA^−Sia^/PA^+Sia^/sialidase-treated PA^+Sia^ at 37°C for 30 min. As before, anti-siglec antibodies were used to block siglecs present on murine/human macrophages. Incubation was followed by washing to remove unbound bacteria. In order to distinguish between surface bound and internalized FITC-PA, cells were briefly treated with Trypan blue (0.02%) to quench FITC fluorescence coming from bacteria which were adherent on the surface. Thus, fluorescence associated with infected macrophages represents phagocytosed PA and was measured by flow cytometry as before.

### Detection of Reactive Oxygen Species (ROS)

Macrophages (1 × 10^6^) were infected with 10 MOI of PA^−Sia^/PA^+Sia^/sialidase-treated PA^+Sia^ and incubated for 30 min at 37°C. Non-adherent bacteria were removed by washing and cells were incubated with a ROS sensitive dye-CM-H_2_DCFDA (10 μM, C6827, Molecular Probes, Invitrogen) for an additional 30 min at 37°C. Cells were washed and analyzed by flow cytometry to estimate ROS generation.

### Quantitation of Nitric Oxide (NO)

Reactive nitrogen species (RNS) represented by NO, is another component of respiratory burst generated by macrophages in response to foreign particles. Intracellular nitric oxide was estimated using DAF-FM diacetate dye. As before, macrophages (1 × 10^6^) infected with PA^−Sia^/PA^+Sia^/sialidase-treated PA^+Sia^ were incubated with DAF-FM (100 nM, D23844, Invitrogen) for 30 min at 37°C. Cells were washed and analyzed by flow cytometry.

### Determination of Secreted Cytokines

J774A.1 cells were activated using PMA (100 ng/mL) (20). These cells (1 × 10^6^) were infected with bacteria (10 MOI) for 45 min at 37°C to allow internalization. Unbound bacteria were washed off. Infected macrophages were further incubated for 3 h at 37°C at 5% CO_2_. Culture supernatants were used to estimate levels of secretory cytokines-IL-10 (555252, BD Biosciences), IL-4 (555232, BD Biosciences), TGF-β (BMS608-4, Invitrogen), and IFN-γ (551866, BD Biosciences) using ELISA kits.

### Intracellular Survival of PA by Gentamicin Protection Assay

Intracellular survival and persistence of PA were assessed by gentamicin assay. Macrophages before and after treatment with anti-siglec antibodies were incubated with PA^−Sia^/PA^+Sia^/sialidase-treated PA^+Sia^ at 10 MOI for 30 min at 37°C. Unbound bacteria were removed by washing. Infected macrophages were further incubated for 3 h with gentamicin (200 μg/mL, G1397, Sigma-Aldrich) in IMDM medium to kill extracellular or surface adherent bacteria. To check the viability of internalized PA, macrophages were lysed in 1% w/v saponin solution. Lysate was serially diluted and spread on tryptic soy agar plates followed by overnight incubation at 37°C. Viable and persistent PA gave rise to colonies and colony forming units (cfu) were used to calculate the number of viable bacteria inside macrophages.

### Intracellular Proliferation of PA by CFSE Staining

Overnight cultured PA^−Sia^ and PA^+Sia^ were pelleted, washed with PBS and enumerated by spectrophotometry. Equal numbers of bacteria were incubated with CFSE dye (50 μM, C34554, Invitrogen) at 37°C for 45 min to ensure uniform staining. Bacteria were repeatedly washed with PBS and then incubated with J774A.1 macrophages at 10:1 ratio at 37°C for 30 min. Unbound bacteria were removed by PBS wash. Remaining uninternalized bacteria were killed by incubation with 200 μg/mL gentamicin. Following 8 h after incubation, cells were visualized by confocal microscopy to detect fluorescence from internalized CFSE stained bacteria.

### Flow Cytometry Based Detection of Bacterial Localization in the Endocytic Pathway

J774A.1 (1 × 10^6^) were incubated with 1:10 MOI of FITC-PA^−Sia^ or PA^+Sia^ for 15 min at 4°C. Plates were then transferred to 37°C to synchronize bacteria uptake and incubated for 20, 40, and 60 min. These cells were scraped off into a phagosome isolation buffer (20 mM HEPES, 0.5 mM EGTA, 0.25 M sucrose, 0.1% gelatin, protease inhibitor cocktail from Calbiochem, Merck-Millipore) (21). Cells were disrupted by passaging (15–20 times) through a 23-gauge needle fitted syringe. The suspension was centrifuged at 200 g for 10 min to separate most of the nucleus and intact cells. Phagosomes were collected by centrifugation of the supernatant at 13,000 g for 5 min and incubated for 5 min with 0.2% saponin, 10% normal goat serum in RPMI. Different endosomal compartments were stained by incubating with primary antibodies against Rab5 (1:100, #12666, Cell Signaling Technology), Rab7 (1:100, #12666, Cell Signaling Technology), and LAMP1 (1:100, #3243, Cell Signaling Technology) in the same buffer for 2 h at 4°C. Phagosomes were counterstained with Alexa Fluor-647 tagged secondary antibodies (A-21245, Invitrogen). The localization of PA in various endosomal compartments was detected by flow cytometry (22).

Additionally, macrophages were infected for 45, 90, and 120 min, labeled with Lysotracker red dye and processed similarly as described above. The colocalization of FITC-PA into Lysotracker red positive lysosomal compartment was detected by flow cytometry.

### Intracellular Calcium Concentration Estimation by Fluorescence Assay

J774A.1 (1 × 10^4^) in microwell plates were incubated with Fura-2-AM (10 μM, Sigma-Aldrich) in presence of 0.04% pluronic-F in Hank's balanced salt solution (HBSS, pH 7.4) containing calcium (CaCl_2_ 0.14 g/L) at 37°C for 30 min. Additionally, few cells were treated with ionomycin (10 μM) to estimate Rmax (high calcium). Similarly, cells were also incubated in calcium-free HBSS and treated with EGTA (10 mM) to estimate Rmin (low calcium). Fura-2-AM stained cells were infected with PA^−Sia^ or PA^+Sia^ at MOI 10 at 37°C and intracellular calcium ions were detected by microplate fluorescence readers detecting fluorescence at 510 nm upon excitation both at 340 and 380 nm. Intracellular calcium concentrations in infected cells were estimated using reference wells as per formula developed by Grynkyewicz (23). Fluorescence readings were taken in Synergy™ 2 Multi-Mode Microplate Reader (Biotek Instruments Inc.).

### Evaluation of Phagosome-Lysosome Fusion in Macrophages Upon Increase in Extracellular or Intracellular Calcium

J774A.1 macrophages were incubated in calcium-free Hank's balanced salt solution (HBSS) buffer or with additional 2.5, 5, 10 mM Ca^2+^ (as CaCl_2_). These cells were infected with 10 MOI of PA^−Sia^/PA^+Sia^ at 4°C for 30 min for bacterial binding followed by transfer to 37°C. Cells were incubated for 30 min to allow phagocytosis in presence of extracellular calcium and then gentamicin (200 μg/mL) was added. Cells were incubated till 3 h followed by quantification of viable internalized bacteria by serial dilution and plating.

Additionally, J774A.1 cells incubated in calcium-free Hank's balanced salt solution (HBSS) buffer were infected with PA^+Sia^ at 10 MOI for 30 min at 4°C. Plates were transferred to 37°C followed by addition of 5 mM Ca^2+^ (as CaCl_2_), ionomycin (10 μM) and were incubated further for 90 min to allow phagocytosis. Macrophages were also stained with Lysotracker red (100 nM) and processed for phagosome purification and flow cytometry as mentioned earlier (22). Macrophages were also prepared for confocal microscopy to detect phagosome lysosome fusion as noted before.

### Immunoblot and Immunoprecipitation Analysis

J774A.1 macrophages (1 × 10^6^) were infected with PA^−Sia^ or PA^+Sia^ at 10 MOI at 37°C for 15, 30, and 45 min, respectively. Infected cells were scraped into PBS, sonicated in presence of protease and phosphatase inhibitor cocktail (Calbiochem, Merck -Millipore) and protein was estimated by BCA assay (Pierce, Thermo Scientific). Equal amounts of protein were resolved in SDS-PAGE (10%) gel and transferred on to a PVDF membrane via wet transfer method (24). Membranes were blocked and incubated with antibodies (1:1,000) against different signaling proteins for overnight. Membranes were next washed using Tris-buffered Saline containing 0.1% Tween-20 (TBS-T) then incubated with appropriate HRP-conjugated secondary antibodies and visualized using chemiluminescence substrate (Pierce, Thermo Scientific) in a Chemidoc imaging system (BioRad Laboratories, CA, USA). The primary antibodies used includes—calmodulin (#4830), calcineurin A (#2614), PI3K-p85 (#4257), PI3K-p110γ (#4252), p-p38 MAPK (#4511), p38 MAPK (#9212), p-Akt Ser473 (#4060), pan-Akt (#4691), p-ERK1/2 (#4377), ERK1/2 (#4695), p-JNK/SAPK (#4668), JNK/SAPK (#9252), β-Actin (#4970), Lamin B (#13435), NF-κB Pathway Sampler Kit (#9936) from Cell Signaling Technology; biotinylated-anti phosphotyrosine (309304) from Biolegend (San Diego, CA, USA); calmodulin-dependent kinase type II (611293), SHP-1 (610126), SHP-2 (610622) from BD Biosciences; siglec-E (sc-377477) from Santacruz Biotechnology.

Additionally, cell lysates were overnight incubated with antibodies against phospho-tyrosine proteins, SHP-1 or SHP-2 phosphatases. Proteins were immunoprecipitated from the lysate using avidin-agarose beads (Invitrogen) or protein-A–sepharose 4B beads (Sigma-Aldrich). Beads were washed with chilled PBS to remove unbound proteins. Protein complexes bound to the beads were solubilized in sample buffer, resolved by SDS-PAGE, transferred to membrane and probed by western blotting process. Membranes were stripped off using stripping buffer (100 mM β-mercaptoethanol, 2% SDS, 62.5 mM Tris-HCl pH 6.8 for 30 min at 50°C) and reprobed using the same antibody used for pulldown to check for protein loading.

Furthermore, cells were suspended into cytosol isolation buffer (10 mM Tris-Cl, 10 mM NaCl, 1.5 mM MgCl_2_, 1 mM PMSF, 0.05% NP-40, pH 6.8), vortexed to allow partial lysis of membranes, centrifuged at 1,000 g for 5 min to precipitate nuclear fractions and cytosol was collected as supernatant. The pellet was washed with chilled PBS, incubated in nuclear isolation buffer (20 mM Tris-Cl, 137 mM NaCl, 1 mM CalCl_2_, 1 mM MgCl_2_, 1 mM PMSF, 1% NP-40, pH 8.0) for 30 min. Nuclear fraction was collected after centrifuging the suspension at 1,000 g for 5 min at 4°C. Both cytosolic and nuclear fractions were similarly resolved via SDS-PAGE and probed by western blotting.

Densitometric analysis of bands was performed using ImageJ software and values normalized with respect to β-actin band intensity have been represented as bar diagrams.

### Knockdown of Siglec-E by siRNA Transfection

J774A.1 cells were transiently transfected with short interfering RNA (siRNA) designed against murine siglec-E (sc-153462, Santacruz Biotechnology) using Lipofectamine and Plus reagent (Invitrogen) as per manufacturer's instructions (15). Silencing of siglec-E was verified by western blotting. Siglec-E silenced J774A.1 cells were similarly infected with PA^+Sia^ and macrophage response to infection was analyzed.

### Statistical Analysis

The data represented are mean values derived from at least three independent experiments. Statistical analysis was performed using the two-tailed Student's *t*-test for two groups of samples and one-way analysis of variance (ANOVA) for more than two groups followed by pair wise multiple comparison procedures using Tukey test with *p* < 0.05 deemed statistically significant. Error-bars represent mean ± standard error of the mean (SEM) from three independent experiments. Significant differences were set at ^ns^*p* > 0.05, ^*^p ≤ 0.05, ^**^p ≤ 0.01, ^***^p ≤ 0.001, and ^****^p ≤ 0.0001 and analyzed by GraphPad Prism version 6.01.

## Results

### Sialylated PA Shows Higher Binding With Murine and Human Macrophages Through Sialic Acid-Siglec Interactions

We routinely confirmed the presence of sialic acid in PA before each experiment by sialic acid specific SNA, MAA lectin binding, and acetyl acetone based fluorimetric estimation of sialic acids.

**Figure 1:**
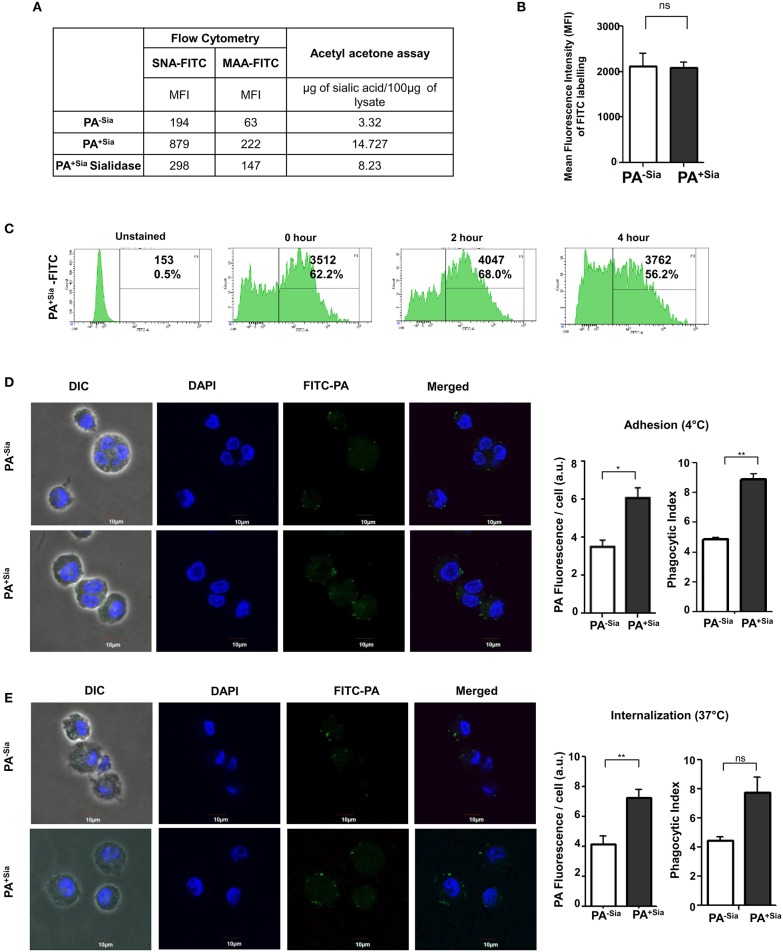
Higher binding and internalization of PA^+Sia^ by J774A.1 macrophages. **(A)** Sialylation of PA was verified using sialic acid specific SNA, MAA lectins and fluorimetric acetyl acetone based sialic acid quantification. Overnight cultured PA (1 × 10^7^) in absence (PA^−Sia^) or presence of serum (PA^+Sia^) were incubated with FITC labeled SNA or MAA lectin (0.02 μg) for 30 min at 4°C, then washed with PBS and analyzed using a flow cytometer. Sialidase PA^+Sia^ were also used in binding assay to confirm presence of sialic acids. MFI values are reported in the table. Additionally, 500 μg of lysates from PA^−Sia^/PA^+Sia^/PA^+Sia^ sialidase were analyzed by fluorimetric acetyl acetone method [*as per* (16)] to quantify presence of sialic acids. The estimated sialic acid content is also shown in the table. **(B)** Equal numbers of bacteria (1 × 10^9^) were incubated with 0.1% FITC in bicarbonate buffer for 45 min at 37°C followed by extensive washing with PBS to remove unbound FITC. These FITC-tagged PA^−Sia^/PA^+Sia^ were then analyzed by flow cytometry to detect extent of labeling. The labeling results from three independent experiments are shown as bar diagram indicating non-significant difference in the labeling of PA in presence and absence of sialic acids. **(C)** After FITC labeling, FITC-PA^+Sia^ was incubated in PBS at 37°C for zero, 2 or 4 h and fixed using 2% p-formaldehyde. Labeled bacteria were analyzed by flow cytometry to detect signal from the FITC tag. Histograms at different time points are provided indicating stable FITC signal upto 4 h. **(D)** FITC-PA^−Sia^/PA^+Sia^ was incubated with J774A.1 macrophages (5 × 10^4^ on coverslips) at 10 MOI (Multiplicity of infection) for 30 min at 4°C to allow adhesion. Non-adherent bacteria were removed, macrophages were stained with DAPI. Images were captured by Andor Spinning Disc Confocal microscope. Green and blue colors represent FITC-bacteria and nuclei, respectively. Average values of green fluorescence associated with macrophages as well as phagocytic indices are calculated using Fiji and represented by a bar diagram. **(E)** Similarly J774A.1 were incubated with FITC-PA^−Sia^ /PA^+Sia^ at 37°C to allow for bacterial internalization. Cells were similarly stained and visualized. Bacterial internalization was estimated from phagocytic index as well as FITC fluorescence associated with macrophages and represented by a bar diagram. Images were representative of three independent experiments. Scale bar = 10 μm. Significance represented by *p ≤ 0.05 and **p ≤ 0.01.

Lectin binding was always higher with the sialylated (PA^+Sia^) than non-sialylated bacteria (PA^−Sia^) indicating presence of sialic acids in PA^+Sia^. This was further corroborated by sialidase treatment which abrogates this binding with PA^+Sia^. The MFI values of lectin binding have been presented in Figure 1A.

Additionally, sialic acid content from PA^−Sia^/PA^+Sia^/PA^+Sia^-sialidase was measured by fluorimetric acetyl acetone method (16). The estimated sialic acid content was always higher in case of PA^+Sia^ as shown in Figure 1A.

Bacteria (PA^−Sia^/PA^+Sia^) were labeled using FITC and analyzed by flow cytometry to confirm extent of labeling. No significant difference in labeling was observed between PA^−Sia^ and PA^+Sia^ despite presence of additional surface sialic acids (Figure 1B). FITC tag was also found to remain stable till 4 h post-labeling (Figure 1C).

Phagocytosis of FITC-PA^+Sia^ and FITC-PA^−Sia^ bacteria by human monocytic (THP-1), as well as murine (J774A.1) macrophage cell lines, were investigated. Phagocytosis involves the recognition and binding of the bacteria to macrophage surface through interactions between bacterial ligands and cellular receptors. This is followed by actual physical internalization by the phagocytic cell (9).

Association between J774A.1 macrophage and FITC-PA^−Sia^/PA^+Sia^ at 4°C was visualized by confocal microscopy (Figure 1D). Higher numbers of FITC-PA^+Sia^ (with phagocytic index = 8.875 ± 0.37) were found to be adherent on macrophage surfaces compared to PA^−Sia^ (4.855 ± 0.14, *p* ≤ 0.01). Images were analyzed to quantitate average FITC fluorescence associated with each macrophage. On an average, macrophages incubated with PA^−Sia^ showed lower fluorescence signal (3.504 ± 0.35 a.u.) as compared to those incubated with PA^+Sia^ (6.067 ± 0.54 a.u., *p* ≤ 0.05).

To check for the internalization of PA, macrophages were incubated with FITC-PA^−Sia^/PA^+Sia^ at 37°C for 30 min and subsequently visualized by confocal microscopy (Figure 1E). A greater number of FITC-PA^+Sia^, represented by green dots (phagocytic index = 7.775 ± 1.02) were visualized inside macrophages, compared to PA^−Sia^ (4.480 ± 0.23). Fluorescence quantitation reveals similar results (macrophages with PA^−Sia^ = 4.178 ± 0.54 a.u., macrophages with PA^+Sia^ = 7.263 ± 0.53 a.u., *p* ≤ 0.01). Thus, macrophages phagocytose more of PA^+Sia^ compared to PA^−Sia^.

**Figure 2:**
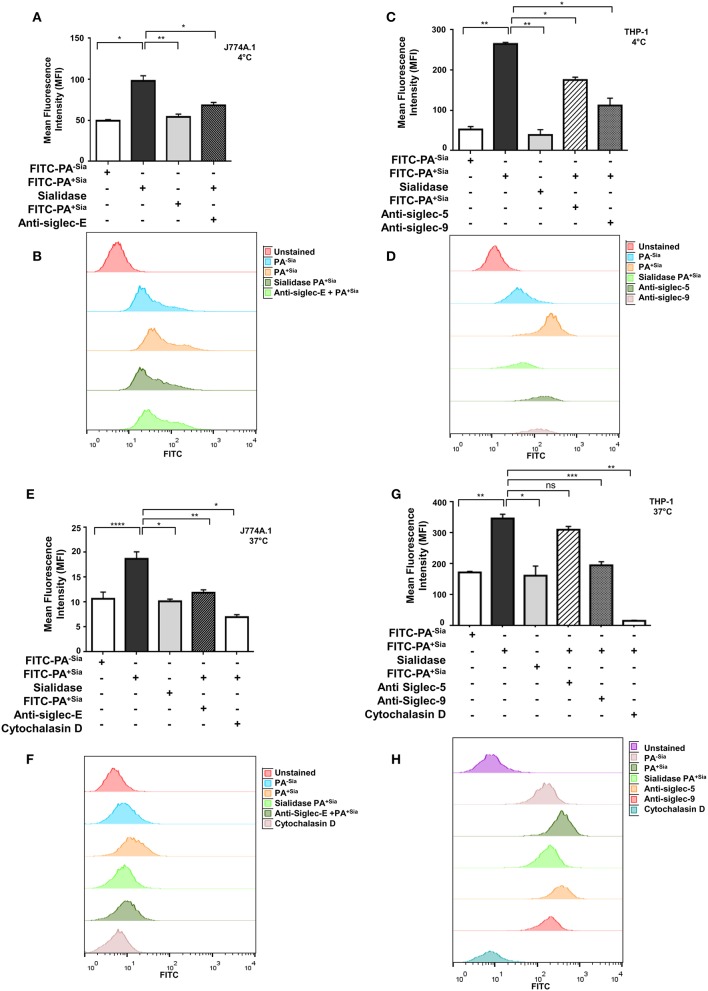
Sialic acid-siglec interactions mediate higher binding of PA^+Sia^ to murine J774A.1 and human THP-1 derived macrophages. **(A,C)** FITC-PA^−Sia^/PA^+Sia^/sialidase treated PA^+Sia^ were incubated with macrophages at 10 MOI for 30 min at 4°C. Macrophages pretreated with blocking antibodies were also incubated with 10 MOI of PA^+Sia^ for 30 min at 4°C. Adhesion of FITC-PA to macrophages was analyzed by flow cytometry. **(B,D)** Representative histograms for the binding assays are provided. **(E,G)** Macrophages were separately incubated at 37°C with FITC-PA^−Sia^/PA^+Sia^/sialidase treated PA^+Sia^. Similarly, blocking antibodies were used to identify receptors involved in the internalization of bacteria by macrophages. Cytochalasin D treatment of macrophages resulted in inhibition of bacterial uptake by phagocytosis. **(F,H)** Representative histograms for internalization assays are shown. The internalization of FITC-PA into macrophages were then analyzed by flow cytometry. MFI values were represented by mean ± s.e.m. from three independent experiments. Significance represented by *p ≤ 0.05, **p ≤ 0.01, ***p ≤ 0.001, and ****p ≤ 0.0001.

Bacterial binding and internalization by macrophages were also analyzed by flow cytometry (Figure 2). As observed in microscopy, binding of FITC-PA^+Sia^ to J774A.1 was significantly higher (~1.5-fold) with mean fluorescence intensity (MFI) values of 90.67 ± 0.88, *p* ≤ 0.05 compared to PA^−Sia^ (59.67 ± 0.88; Figure 2A).

Additionally, PA^+Sia^ were treated with sialidase to remove sialic acids. Desialylation of PA^+Sia^ significantly abrogates (MFI = 63.67 ± 1.45, *p* ≤ 0.01) the higher binding seen in interactions of untreated PA^+Sia^ with J774A.1 (MFI = 90.67 ± 0.88; Figure 2A). Thus, sialic acids on bacteria can be considered as ligands responsible for the differential binding between PA^+Sia^ and PA^−Sia^ to macrophages.

A similar trend was observed when THP-1 derived macrophages were used for binding (Figure 2C). Association of PA^+Sia^ with these macrophages (MFI = 322.7 ± 5.20, *p* ≤ 0.01) was significantly higher compared to PA^−Sia^ (MFI = 85.67 ± 2.60). Also, removal of sialic acids significantly reduces the binding of PA^+Sia^ (MFI = 97.33 ± 6.17, *p* ≤ 0.01).

Sialic acids are recognized and bound by siglecs on host cells. In order to identify particular siglecs responsible for the increased association of PA^+Sia^ with macrophages, cells were pretreated with blocking antibodies against siglecs. Flow cytometry data revealed that blocking of siglecs on J774A.1 macrophages with anti-siglec-E antibodies results in significant reduction in PA^+Sia^ binding (MFI = 63.00 ± 0.57, *p* ≤ 0.05) as compared to untreated macrophages (MFI = 90.67 ± 0.88; Figure 2A).

Binding of PA^+Sia^ to THP-1 derived macrophages (MFI = 322.7 ± 5.20) was similarly reduced when siglecs were blocked by anti-siglec 9 antibodies (MFI = 148.7 ± 4.05, *p* ≤ 0.05; Figure 2C). Treatment of macrophages with anti-siglec-5 antibody also slightly reduces the binding of PA^+Sia^ (MFI = 272.7 ± 3.18, *p* ≤ 0.05). Representative histograms for the binding assays with J774A.1 cells are also shown in Figure 2B and with THP-1 is shown in Figure 2D. Blocking of other siglecs, namely murine siglec-1 and human siglecs-1, 3, and 7 show minute changes in PA binding (Supplementary Figure S1A).

Few other macrophage receptors are also known to mediate non-opsonic phagocytosis of PA (11). Contribution of such receptors in binding of PA^+Sia^ was also checked by using anti-CD11b (CR-3 alpha chain) and anti-CD16/32 (FcγRII and FcγRIII) antibodies. Although these macrophage receptors are also involved in binding, siglecs also play an important role in PA^+Sia^ binding with macrophages (Supplementary Figure S1A).

In similar experimental set up, macrophages were incubated with FITC-PA at 37°C for 30 min to allow for bacterial uptake, which was measured by flow cytometry (Figures 2E,G). PA^+Sia^ was internalized more (MFI = 54.33 ± 0.33, *p* ≤ 0.0001) by J774A.1 compared to PA^−Sia^ (MFI = 42.00 ± 0.57). This higher internalization was abrogated when sialic acid was removed from PA^+Sia^ (MFI = 42.33 ± 0.33, *p* ≤ 0.05; Figure 2E).

Furthermore, blocking of siglecs on J774A.1 by anti-siglec-E antibodies also reduced PA^+Sia^ phagocytosis (MFI = 41.00 ± 1.000, *p* ≤ 0.01) suggesting sialic acid and siglecs interactions are responsible for this higher internalization (Figure 2E). Representative histogram for the internalization assay with J774A.1 is shown as Figure 2F.

THP-1 derived macrophages also show similar pattern of interaction with PA (Figure 2G). Macrophages phagocytose more of PA^+Sia^ (MFI = 352.7 ± 5.78, *p* ≤ 0.01) compared to PA^−Sia^ (MFI = 107.3 ± 3.52). Also, desialylation of PA^+Sia^ abolishes this higher internalization (MFI = 116.3 ± 4.66, *p* ≤ 0.05; Figure 2G). Moreover, anti-siglec-9 antibodies treatment of THP-1 derived macrophages exhibited reduced bacterial internalization (MFI = 147.7 ± 4.05, *p* ≤ 0.001) indicating sialic acid-siglec-9 interaction to be majorly responsible for higher phagocytosis of PA^+Sia^ compared to siglec-5 (Figure 2G). As before, representative histogram for the internalization assay with THP-1 cells is shown as Figure 2H.

In order to further verify that PA entry into macrophages is due to its uptake by macrophages, cells were treated with cytochalasin D, which inhibits actin polymerization. Cytochalasin D nearly abolished bacterial uptake in both J774A.1 (MFI = 16.50 ± 0.50, *p* ≤ 0.05) and THP-1 derived macrophages (MFI = 23.33 ± 2.40, *p* ≤ 0.01; Figures 2E,G) confirming that macrophages are actively internalizing bacteria.

Taken together, these results demonstrate that PA^+Sia^ binding to macrophages is mediated through sialic acid-siglec interactions mainly through siglec-E and siglec-9 subsequently leading to higher phagocytosis.

### Internalization of PA^+Sia^ by Macrophages Results in Poor Bactericidal Responses and Altered Cytokine Response

**Figure 3:**
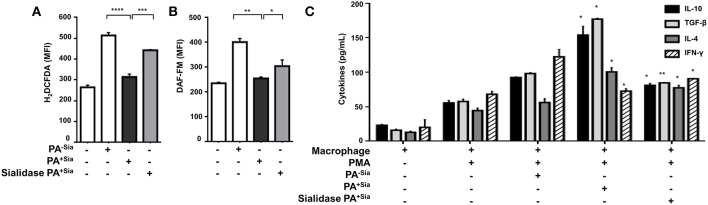
PA^+Sia^-infected macrophages exhibit a reduced bactericidal response. **(A)** J774A.1 (1 × 10^6^/well) were infected similarly with PA^−Sia^/PA^+Sia^/sialidase treated PA^+Sia^ and then stained with CM-H_2_DCFDA. Intracellular ROS production in uninfected and infected macrophages was estimated by flow cytometry. Data were analyzed by FACSDiva 8.0.2 software **(B)** These cells were additionally stained with DAF-FM. Intracellular RNS production was then estimated. Data were analyzed as before. **(C)** Following PMA stimulation, J774A.1 cells were infected with PA^−Sia^/PA^+Sia^ for 3 h. Culture supernatants were collected and levels of IL-10, IL-4, TGF-β, and IFN-γ secreted by macrophages were quantified using ELISA kits as per manufacturer's instructions. All data represented were from three independent experiments (mean ± s.e.m.). Significance represented by *p ≤ 0.05, **p ≤ 0.01, ***p ≤ 0.001, and ****p ≤ 0.0001.

J774A.1 were infected with PA^−Sia^ or PA^+Sia^ for 30 min at 37°C followed by staining with CM-H_2_DCFDA, an indicator dye for cellular ROS (Figure 3A). PA^+Sia^ infected macrophages were found to exhibit significantly lowered ROS levels (MFI = 314.5 ± 7.81, *p* ≤ 0.0001) compared to PA^−Sia^ infected macrophages (MFI = 513.7 ± 8.24). Interestingly, removal of sialic acids from PA^+Sia^ resulted in enhancement of ROS production in macrophages (MFI = 442.7 ± 1.20, *p* ≤ 0.0001).

Similarly, intracellular levels of nitric oxide generated were measured by staining infected macrophages with DAF-FM (Figure 3B). It was observed that macrophages infected with PA^+Sia^ show lower levels of intracellular nitric oxide (MFI = 255.2 ± 2.85, *p* ≤ 0.01) compared to PA^−Sia^ infected macrophages (MFI = 401.6 ± 10.10). Removal of sialic acids from PA^+Sia^ also resulted in enhanced generation of nitric oxide (MFI = 304.4 ± 18.10, *p* ≤ 0.05).

Furthermore, we compared the modulation of cytokine response in PA^−Sia^ or PA^+Sia^ infected macrophages (Figure 3C). Macrophages were stimulated with PMA and culture supernatant from stimulated uninfected cells were used to establish baseline of cytokine secretion. Culture supernatants from stimulated macrophages infected with PA^+Sia^ show further elevation in levels of TH2 type cytokines (IL-10 = 154.0 ± 9.00 pg/mL, TGF-β = 177.0 ± 1.00 pg/mL, IL-4 = 100.5 ± 4.50 pg/mL) and reduced secretion of IFN-γ (77.50 ± 2.50 pg/mL). In contrast, macrophages infected with PA^−Sia^ showed comparatively reduced levels of TH2 cytokines (IL-10 = 92.50 ± 0.50 pg/mL, TGF-β= 98.00 ± 1.00 pg/mL, IL-4 = 56.00 ± 4.00 pg/mL,) and slightly higher secretion of pro-inflammatory cytokine IFN-γ (122.5 ± 7.50 pg/mL).

Removal of sialic acids from PA^+Sia^ reversed the cytokine profile leading to lowered secretion of TH2 type cytokines (IL-10= 81.00 ± 2.00 pg/mL, TGF-β= 84.50 ± 0.50 pg/mL, IL-4= 77.50 ± 2.50 pg/mL) and increased IFN-γ secretion (90.50 ± 0.50 pg/mL) suggesting an immunosuppressive role of sialic acid.

Thus, it may be noted that reduced respiratory burst and anti-inflammatory cytokine secretion in PA^+Sia^-infected macrophages, can be reversed by desialylation of PA^+Sia^, suggesting that sialic acids-siglec interactions may play a role in this altered immune response.

### Phagocytosed PA^+Sia^ Evades Phagosome-Lysosome Fusion and Remain Viable Inside Macrophages

So far, we have observed that higher numbers of PA^+Sia^ are internalized by macrophages relative to PA^−Sia^. However, attenuation of respiratory burst and anti-inflammatory cytokine responses by macrophages will have a negative impact on bacterial killing. Thus, the clearance of phagocytosed PA^+Sia^ depends on the endocytic pathway, specifically phagosome maturation and phagolysosome formation.

**Figure 4:**
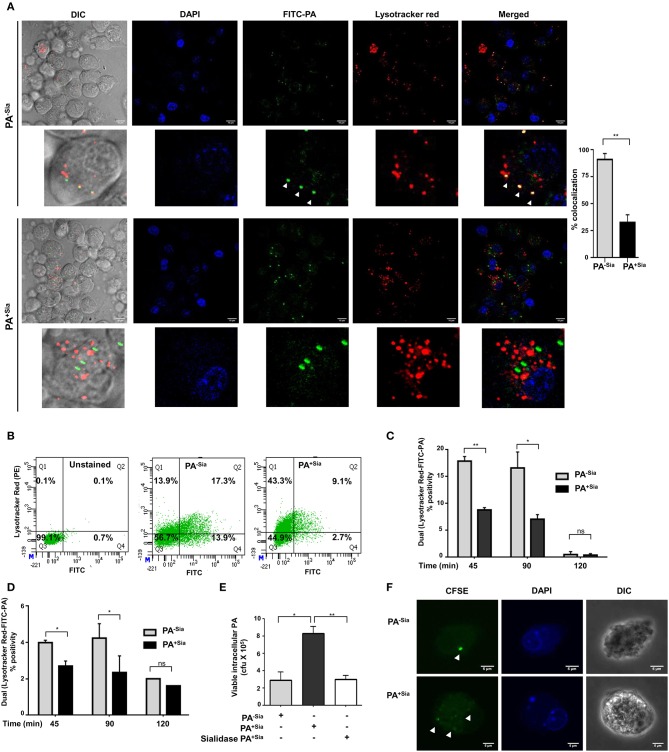
Phagosome-lysosome fusion was impaired in PA^+Sia^ infected macrophages leading to bacterial survival. **(A)** J774A.1 cells (5 × 10^4^) seeded on coverslips were infected with FITC-PA^−Sia^/PA^+Sia^ at MOI 10 for 15 min at 4°C, non-adherent bacteria were then removed and infection was continued till 1 h at 37°C. Lysosomes were labeled with Lysotracker red dye, cells were fixed, mount and analyzed by microscopy. Colocalization of bacteria (green) and lysosomes is indicated by yellow color in merged images. Also, white arrows are used to denote areas of colocalization between FITC-PA and lysosomes. Images are representative of three independent experiments performed. Zoomed-in images are also provided. Scale bar = 10 μm. **(B,C)** Fusion of phagosomes with lysosomes was analyzed through flow cytometry. Lysotracker stained J774A.1 were similarly infected with FITC-PA^−Sia^/PA^+Sia^ for 45, 90, and 120 min. Cells were gently lysed, phagosomes were collected and analyzed by flow cytometry. Dual fluorescence positivity represents successful phagosome-lysosome fusion as shown by dot plots and bar graphs. Bar graphs are prepared from data (mean ± s.e.m.) of three independent experiments. **(D)** Phagosome-lysosome fusion was similarly quantified in human macrophages (THP-1 derived macrophages) and shown in a bar graph. Data represented (mean ± s.e.m.) from three independent experiments. **(E)** Viability of bacteria in macrophages following phagocytosis was assessed using gentamicin protection assay. Macrophages were incubated with PA^−Sia^/PA^+Sia^/sialidase-treated PA^+Sia^ at 1:10 MOI for 30 min at 37°C followed by 3 h incubation with gentamicin (200 μg/mL). The viability and persistence of internalized bacteria were checked by plating the lysed macrophage contents on nutrient agar plates and checking for growing bacterial colonies (colony forming units, cfu). Data represented as mean ± s.e.m. of three independent experiments. **(F)** Macrophages infected with CFSE stained PA^−Sia^/PA^+Sia^ were visualized by confocal microscopy to detect presence of bacteria (green specks). Scale bar = 5 μm Data represented as mean ± s.e.m. of three independent experiments. In all experiments, significance is represented as ^ns^*p*> 0.05, *p ≤ 0.05, and **p ≤ 0.01.

Accordingly, we checked the final fate of PA^+Sia^ phagosomes inside the macrophages (Figure 4A). FITC-PA^−Sia^ or PA^+Sia^ infected J774A.1 macrophages were stained with Lysotracker red. Confocal microscopy revealed that across multiple fields, more numbers of green dots (intracellular PA^−Sia^) colocalized with the red lysosomes giving a composite yellow color in the merged fields in contrast to PA^+Sia^-infected macrophages. Quantification of colocalization revealed that 98.7% of PA^−Sia^ containing phagosomes colocalize with the lysosomal compartment while only 31.7% of PA^+Sia^ containing phagosomes were found to do so.

We used flow cytometry to further quantify phagosome-lysosome fusion. Lysotracker red stained macrophages infected with FITC-PA^−Sia^ or PA^+Sia^ for 45, 90 and 120 min were gently lysed and released phagosomes were analyzed in a flow cytometer. Out of all phagosomes, lysosomes and phagolysosomes were identified by incorporated lysotracker stain. The presence of FITC-PA in lysosomes, signifying phagosome-lysosome fusion, was quantified by dual color (FL1-FL2 channel) positivity (Figure 4B).

In infected J774A.1, we observed that PA^+Sia^ colocalized with lysosomes was lower being only 8.800 ± 0.30% (*p* ≤ 0.01) of all analyzed events compared to PA^−Sia^ (17.90 ± 0.60%) after 45 min of infection (Figure 4C). A similar trend was observed after 90 min of infection. The percentage of dual positive phagosomes diminished at 120 min post infection. We confirmed our observations in THP-1 derived macrophages, which showed a similar kind of colocalization at 45, 90 and 120 min upon infection with FITC-PA^−Sia^/PA^+Sia^ (Figure 4D). Thus, the flow cytometry data corroborates with our observations from confocal microscopy, that fusion of PA^+Sia^-containing phagosomes with lysosomes was reduced.

Earlier, we demonstrated a comparatively lowered oxidative burst and reduced pro-inflammatory cytokines secretion (Figure 3) in PA^+Sia^-infected macrophages compared to PA^−Sia^. Furthermore, phagosome-lysosome fusion, which eliminates phagocytosed bacteria, is also reduced in PA^+Sia^-infected macrophages (Figure 4). Thus, we checked if PA^+Sia^ bacteria remain viable following internalization by macrophages through gentamicin protection assay (Figure 4E). Results indicate that approximately 3.4-fold higher number of PA^+Sia^ survived inside macrophages compared to PA^−Sia^; colony forming units being 8.33 ± 0.43 × 10^5^ vs. 2.90 ± 0.55 × 10^5^ for PA^+Sia^ *vs*. PA^−Sia^ respectively (*p* ≤ 0.05) at 3 h post-infection.

Removal of sialic acids from PA^+Sia^ resulted in ~ >2.6-fold reduction in the number of viable bacteria isolated from infected macrophages. These observations clearly indicate that bacterial sialic acid provide internalized PA^+Sia^ with additional advantage to survive inside macrophages.

So far we have observed higher numbers of viable PA^+Sia^ inside macrophages after 3 h post infection by gentamicin protection assay. Staining of bacteria with CFSE is one of the methods to check bacterial multiplication inside phagocytic cells (25–27). Therefore, to check if internalized PA^+Sia^ can replicate inside macrophages, J774A.1 macrophages were infected with CFSE stained PA^−Sia^/PA^+Sia^.

Macrophages infected for 8 h were visualized by confocal microscopy to check for the presence of PA (Figure 4F). While only a few PA^−Sia^ bacteria were observed as bright green dots inside the cells, multiple dots of faintly glowing PA^+Sia^ were visible. This suggests that CFSE stained PA^+Sia^ undergoes division inside macrophages which resulted in dilution of CFSE dye.

### Reduced Recruitment of Rab7 Results in Inhibition of Phagosome-Lysosome Fusion in PA^+Sia^-Infected Macrophages

Phagosome maturation consists of early, mid and late stage phagosomes. Each stage is characterized by sequential association and dissociation of specific effector Rab proteins (28). The final step always is the fusion of the phagosome with lysosomes subsequently resulting in degradation of its cargo. Since phagosome-lysosome fusion was reduced in PA^+Sia^-infected macrophages, we wanted to identify if any intermediate step in phagosome maturation was hindered by checking for changes in the acquisition of stage-specific Rab proteins.

Previous experiments have demonstrated that internalized PA is trafficked into the lysosomal compartment within 45 min of infection. Accordingly, J774A.1 infected with PA^−Sia^/PA^+Sia^ were fixed at 20, 40, and 60 min after infection, permeabilized and stained for early endosomal (Rab5), late endosomal (Rab7) and lysosome specific (LAMP1) markers, respectively. Cells were counterstained with secondary antibody and visualized by confocal microscopy. Nearly 100 cells from different randomly chosen fields as well as z stacks based visualizations were analyzed for colocalization of internalized bacteria with the endosomal compartments.

**Figure 5:**
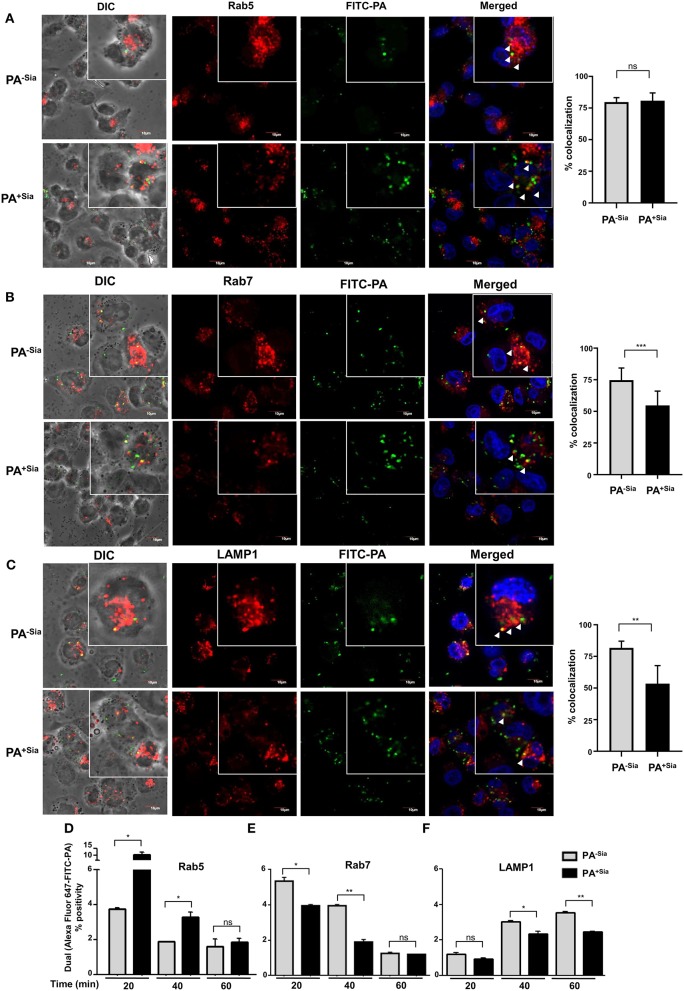
Trafficking of PA^+Sia^ is altered in different endocytic compartments. **(A–C)** Coverslip adherent macrophages were incubated with 1:10 MOI of FITC-PA^−Sia^/ PA^+Sia^ for 15 min at 4°C followed by further incubation for 20, 40, and 60 min at 37°C. Cells were then fixed, permeabilized using PBS containing 0.3% Triton-X. Cells infected for 20, 40, and 60 min were stained with anti-Rab5, Rab7, and LAMP1 antibodies, respectively. Cells were washed, counterstained with Alexa Fluor-647 tagged secondary antibodies and mount, sealed. Images were randomly acquired from multiple fields by confocal microscopy as well as from multiple z stacks. Images were processed and co-localization calculated using Fiji software. Colocalization results are calculated from minimum 100 cells from different fields. White arrows denote areas of colocalization between FITC-PA and labeled endosomes. Scale bar = 10 μm **(D–F)** J774A.1 were similarly infected with FITC-PA^−Sia^/PA^+Sia^ for 20, 40, and 60 min. Cells were then processed as before and phagosomes were collected. Phagosomes were stained for Rab5, Rab7 or LAMP1 and analyzed by flow cytometry. Localization of FITC-PA was analyzed by dual fluorescence positivity. Data represented as mean ± s.e.m. of three independent experiments. In all experiments, significance is represented as ^ns^*p*> 0.05, *p ≤ 0.05, **p ≤ 0.01, ***p ≤ 0.001.

At the initial phase of infection (20 min), 79.67 ± 3.53% of internalized PA^−Sia^ and 80.82 ± 6.15% of internalized PA^+Sia^ were located in Rab5-stained compartment (Figure 5A). Although PA^−Sia^ and PA^+Sia^ were equally localized in the Rab5 labeled early phagosomes, the number of PA^+Sia^ taken up by the macrophages remained higher.

Maturation of early phagosomes into late phagosomes is marked by the appearance of Rab7 protein which is necessary for the final step of phagosome fusion to lysosomes (29). Accordingly, 40 min post-infected macrophages were stained with anti-Rab7 antibody. It was observed that 74.67 ± 9.61% of phagocytosed PA^−Sia^ colocalized within the Rab7 stained compartment after 40 min of incubation (Figure 5B). Interestingly, reduced numbers of PA^+Sia^-containing phagosomes (54.70 ± 11.31%, *p* ≤ 0.001) were found to acquire Rab7. Additionally, we compared colocalization between macrophages that had internalized higher numbers of PA^−Sia^ and PA^+Sia^. Even at comparable numbers, PA^−Sia^ always showed higher colocalization with Rab7 (Supplementary Figure S1B) while PA^+Sia^ evaded fusion with the Rab7 compartment.

Next we wanted to observe successful fusion of PA-containing phagosomes with lysosomes. Accordingly, lysosomes were labeled with anti-LAMP1 antibody in 60 min post-infected macrophages. Higher percentage of PA^−Sia^-containing phagosomes (81.72 ± 5.33%) was found to have reached the lysosomal compartments (Figure 5C). However, at the same time, only 53.46 ± 14.23% (*p* ≤ 0.05) of PA^+Sia^-containing phagosomes were LAMP1 positive.

These observations were validated using flow cytometry based analysis of PA containing phagosomes. FITC-PA^−Sia^/PA^+Sia^ infected J774A.1 were incubated for 20, 40, and 60 min and prepared phagosomal fractions were stained for early endosomal (Rab5), late endosomal (Rab7) and lysosome specific (LAMP1) markers. Flow cytometry revealed that at an early time point of infection (20 min), higher numbers of PA^+Sia^ colocalized with the Rab5 labeled compartment (10.30 ± 1.30%, *p* ≤ 0.05) compared to PA^−Sia^ (3.750 ± 0.05%; Figure 5D). This trend remains even up to 40 min post-infection (PA^+Sia^ = 3.300 ± 0.20%, PA^−Sia^ = 1.900 ± 0.0%, *p* ≤ 0.05). Possibly, the greater presence of PA^+Sia^ in Rab5 positive early phagosomes at early time points is due to its higher phagocytosis by J774A.1. By 60 min post-infection, only a few PA^+Sia^/PA^−Sia^ remain in the Rab5 labeled compartment.

Interestingly, we observed that PA^+Sia^ harboring phagosomes acquire approximately 0.8-fold less Rab7 (Figure 5E). The percentage of PA^+Sia^ phagosomes that were positive for Rab7 were 3.950 ± 0.05 % out of all events at 20 min post infection which was significantly lower compared to Rab7 positive PA^−Sia^ phagosomes (5.350 ± 0.15 %, *p* ≤ 0.05). At 40 min post infection, Rab7-positive phagosomes containing PA^+Sia^ were even less (1.900 ± 0.10%) as compared to PA^−Sia^ (3.950 ± 0.05%, *p* ≤ 0.01).

We also checked for colocalization of bacteria with the LAMP1 labeled compartment, which represents lysosomes and phagolysosomes (Figure 5F). At 20 min, the difference between PA^−Sia^ and PA^+Sia^ phagosomes was low mostly because very few phagosomes had matured at this early time point. We observed that PA^−Sia^ phagosomes associated with LAMP1 at 40 min was higher (3.050 ± 0.05%) than PA^+Sia^ (2.350 ± 0.15%, *p* ≤ 0.05). At 60 min post-infection, a similar trend was observed. Colocalization of PA^−Sia^ to lysosomes was 3.550 ± 0.05% while in case of PA^+Sia^ this value was lower (2.450 ± 0.05, *p* ≤ 0.01).

Taken together, our observations indicate that 20 min after infection, significantly higher numbers of PA^+Sia^ was localized in Rab5 positive early phagosomes than PA^−Sia^. However, at the same time point, more PA^−Sia^ had colocalized with the Rab7 compartment compared to PA^+Sia^. At 40 min after infection, higher numbers of PA^+Sia^ continue to remain associated with early phagosomes while comparatively more PA^−Sia^-phagosomes mature and become Rab7 positive. Simultaneously, the PA^−Sia^ colocalization with LAMP1 was higher than PA^+Sia^, signifying successful fusion to lysosomes. This observation was also confirmed by flow cytometry in FITC-PA^−Sia^ and PA^+Sia^ infected THP-1 derived macrophages (Supplementary Figure S1C).

Thus, our observations indicate that reduced acquisition of Rab7 by phagosomes harboring PA^+Sia^ may be responsible for the subsequent prevention of phagosome-lysosome fusion as demonstrated by the reduced acquisition of LAMP1.

### PA^+Sia^ Modulates the Intracellular Calcium Level of Macrophages and Alters Calcium Related Signaling Thereby Affecting Phagolysosome Fusion

**Figure 6:**
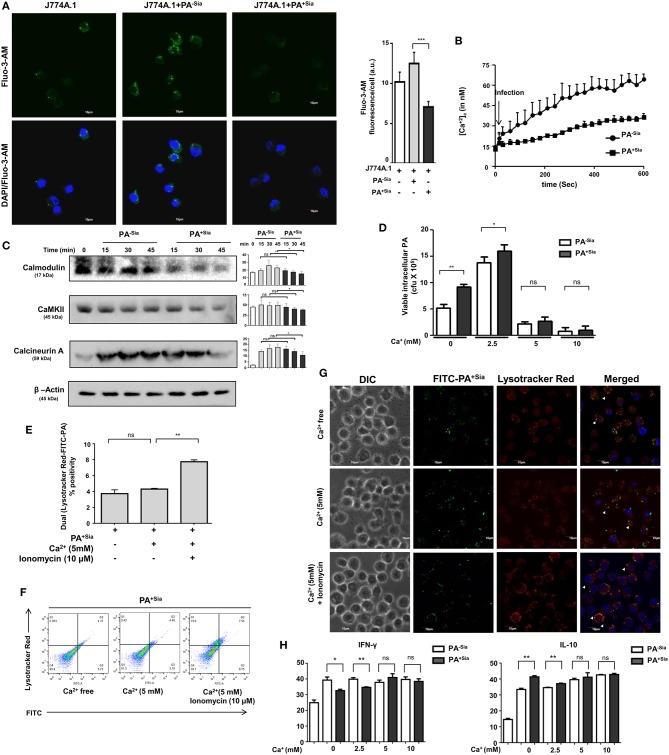
Intracellular calcium concentration and calcium sensing proteins are reduced in PA^+Sia^ infected-J774A.1. **(A)** Fluo-3-AM stained J774A.1 (5 × 10^4^) plated on coverslips were infected with 10 MOI of PA^−Sia^/PA^+Sia^ at 37°C for 15 min, fixed, mounted on slides and visualized using Fluoview fv10i confocal microscope. Green fluorescence represents intracellular calcium levels. Images are representative of three independent experiments. Scale bar = 10 μm. **(B)** Intracellular calcium levels in PA^−Sia^/PA^+Sia^ infected macrophages were also quantified by Fura-2-AM staining and detection of cellular fluorescence in a plate fluorimeter. Fluorescence was converted into calcium concentration using Grynkewicz equation by using reference wells. Data (mean ± s.e.m.) is representative of three independent experiments. **(C)** Expression levels of calcium-sensing proteins (calmodulin, calmodulin kinase type II and calcineurin) were detected in PA^−Sia^/PA^+Sia−^infected macrophages. J774A.1 was infected for 15, 30, and 45 min, lysates were analyzed by western blotting. The expression of β-actin was used to account for equal loading. Western blots **(C)** are representative of three independent experiments. Densitometric values reported are normalized with respect to β-actin band intensities. **(D)** Macrophages in calcium free Hank's balanced salt solution (HBSS) or supplemented with extracellular Ca^2+^ at 2.5, 5, 10 mM concentration were incubated with PA^−Sia^/PA^+Sia^ at 4°C for 30 min to allow bacterial binding. Cells were then transferred to 37°C for 30 min to allow phagocytosis and then gentamicin (200 μg/mL) was added. Cells were incubated till 3 h followed by quantification of internalized viable bacteria by plating. **(E,F)** Macrophages were incubated with PA^+Sia^ in presence of enhanced extracellular Ca^2+^ (5 mM) or intracellular Ca^2+^ (5 mM Ca^2+^, 10 μM ionomycin) for 90 min at 37°C. Phagolysosome fusion was detected in phagosomes released from these macrophages by flow cytometry as previously described. The results from three independent experiments are averaged and shown as bar diagrams with statistical significance. Scatter plots to detect double-positive populations are also shown. **(G)** Previous experiment was repeated and infected macrophages were observed by confocal microscopy to detect phagosome-lysosome fusion in infected macrophages in presence of enhanced extracellular or intracellular calcium. Images are representative of three independent imaging that was performed. **(H)** Cytokine response by PA^−Sia^/PA^+Sia^ infected macrophages in presence of extracellular calcium of different concentrations was checked by ELISA. Levels of IFN-γ (pro-inflammatory cytokine) and IL-10 (anti-inflammatory cytokine) were estimated. Significance represented by ^ns^*p* > 0.05, *p ≤ 0.05, and **p ≤ 0.01.

Modulation of intracellular calcium ions during phagocytosis has been linked to evasion of phagosome-lysosome fusion in *Mycobacterium tuberculosis* infections (30). Thus, we next evaluated intracellular calcium levels in PA^−Sia^ or PA^+Sia^-infected macrophages (Figure 6A). Microscopy revealed that intracellular calcium levels, [Ca^2+^]_c_ were lower in Fluo-3-AM loaded PA^+Sia^ infected macrophages (7.075 ± 0.34 a.u., *p* ≤ 0.001) compared to PA^−Sia^ (12.50 ± 0.70 a.u.) at 15 min post infection.

Additionally, intracellular calcium concentrations in Fura-2-AM stained infected macrophages were also analyzed by fluorescence plate reader and concentration of calcium ions (nM) was calculated using the Grynkewicz equation (Figure 6B). Following infection, intracellular calcium concentrations remained lower in PA^+Sia^-infected macrophages as compared to PA^−Sia^.

Alteration of intracellular calcium levels results in modulating a cascade of signaling events in the cytosol. Calmodulin is an important cytosolic calcium-binding protein that is known to regulate phagosome-lysosome fusion in *Mycobacterium* (30, 31). Accordingly, we analyzed the levels of a few calcium-related proteins in infected macrophages by western blotting (Figure 6C). Statistically significant differences were observed in levels of calmodulin, calcineurin, and calmodulin-dependent kinase type II expression only after 45 min post infection in PA^+Sia^ infected macrophages.

So far we have observed that PA^+Sia^ infection of J774A.1 macrophages are associated with lowered intracellular calcium concentrations and calcium-related proteins. As mentioned before, lowered calcium concentrations are correlated with phagosome lysosome fusion inhibition in *Mycobacterium* (30, 31). Normally, infection was carried out in IMDM medium containing 1.5 mM calcium concentration (as CaCl_2_). Gentamicin protection assay was used to determine if addition of extracellular calcium (final concentration −2.5, 5, 10 mM) affects the viability of PA^+Sia^ at 3 h post infection. It was observed that bacterial viability was not affected in Ca^2+^ free condition or at lower concentrations of extracellular calcium. However, viability of PA^−Sia^ and PA^+Sia^ was inhibited onwards from 5 mM extracellular Ca^2+^ concentration (Figure 6D).

We next checked if loss of viability of PA^+Sia^-infected macrophages in 5 mM extracellular Ca^2+^ was due to enhanced phagosome-lysosome fusion. Macrophages in calcium free HBSS were infected with PA^+Sia^ at 4°C for binding. Plates were transferred to 37°C and 5mM extracellular Ca^2+^ ionomycin (10 μM) was added. Phagocytosis was continued in macrophages incubated in calcium-free HBSS or containing Ca^2+^ (5 mM) or both Ca^2+^ (5 mM) and ionomycin for 90 min. Cells were finally labeled with lysotracker red, phagosomes were prepared and analyzed by flow cytometry. Surprisingly, compared to infected macrophages in absence of calcium, addition of only extracellular Ca^2+^ did not greatly increase phagolysosome fusion events (Figure 6E). However, introduction of ionomycin along with extracellular Ca^2+^ (5 mM) resulted in increase in the percentage of phagosome-lysosome fusion events. Ionomycin is known to increase intracellular calcium levels. Representative scatter plots indicating colocalization between lysotracker stained lysosome or phagolysosomes with FITC-PA^+Sia^ are shown (Figure 6F).

Confocal microscopy also confirmed these observations. Phagosome-lysosome fusion remained unchanged at high extracellular Ca^2+^ concentrations (Figure 6G). But, addition of ionomycin increased the intracellular calcium concentration and enhanced phagolysosome fusion in PA^+Sia^ infected macrophages (Figure 6G).

These observations indicate that loss of viability of bacteria in presence of extracellular calcium was not related to phagolysosome fusion. However, in presence of extracellular calcium, ionomycin-mediated rapid increase in intracellular calcium enhanced phagosome-lysosome fusion in PA^+Sia^ infected macrophages. This further confirms that intracellular Ca^2+^ plays a regulatory role in bacterial killing through phagolysosome formation in PA^+Sia^-infected macrophages.

Inflammatory cytokine response by macrophages incubated with PA^−Sia^ or PA^+Sia^ in presence of increased extracellular calcium was also checked by ELISA based estimation of IFN-γ (pro-inflammatory) and IL-10 (anti-inflammatory). No significant change in cytokine secretion was observed with increase in calcium (Figure 6H). Interestingly, the differences in cytokine response between PA^−Sia^/PA^+Sia^-infected macrophages were diminished with increasing Ca^2+^.

### Engagement of Siglec-E by Bacterial Sialic Acids Results in Siglec-Based Inhibitory Signaling in Macrophages

**Figure 7:**
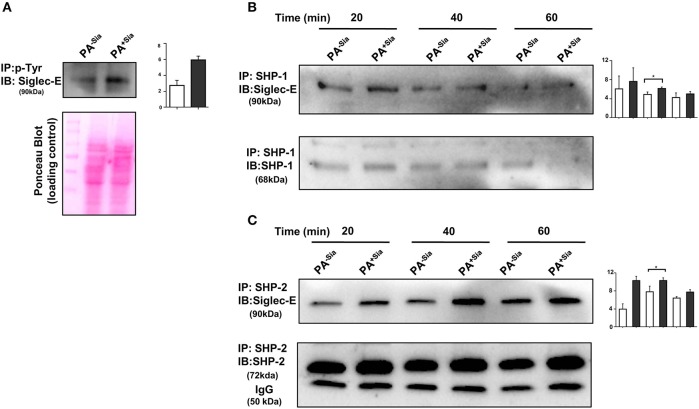
Interaction of siglec-E-sialic acids results in siglec-based inhibitory signaling. **(A)** Phosphorylation of siglec-E was checked by immunoprecipitation of lysates of PA^−Sia^/PA^+Sia^ infected macrophages with biotinylated anti-phosphotyrosine antibodies using avidin-agarose beads. Precipitated proteins were resolved via SDS-PAGE, transferred to PVDF membrane and probed for siglec-E using antibodies. Image of Ponceau S stained membrane has been used to account for equal loading of proteins. **(B,C)** Association between SHP-1 and SHP-2 with siglec-E was quantified by immunoprecipitating infected macrophage lysates with anti-SHP-1 or SHP-2 antibody and protein A- sepharose 4B beads. Precipitated complexes were analyzed by western blotting. All western blots are representative of three independent experiments. Densitometric values reported are normalized with respect to SHP-1/SHP-2 band intensities. Average of normalized band intensities from three independent experiments are represented as bar diagrams with statistical significance.

Binding of sialic acids by siglecs result in phosphorylation of tyrosine residues present in the ITIM motifs of inhibitory siglec-E leading to the recruitment of SHP-1 and SHP-2 phosphatases. We have already demonstrated that PA^+Sia^ binds to J774A.1 through siglec-E (Figure 2). Immunoprecipitation revealed 2.75-fold higher phosphorylation of tyrosine residues of siglec-E in PA^+Sia^-infected macrophages compared to PA^−Sia^, indicating such engagement of siglec-E leads to its activation (Figure 7A). A representative Ponceau blot is provided to account for equal loading across lanes. Next, we analyzed the association between siglec-E and SHP1/2 phosphatases (Figures 7B,C). Higher levels of siglec-E were found to be associated with SHP-2 in macrophages infected with PA^+Sia^ compared to PA^−Sia^ at each time point (20, 40, and 60 min). At 40 min, association between siglec-E and SHP-2 was significantly ~1.5-fold times higher (Figure 7C). These observations suggest that siglec-mediated inhibitory signaling is active in PA^+Sia^-infected macrophages. A similar trend was also observed with SHP-1 (Figure 7B). Association between siglec-E and SHP-1/2 was also significantly higher at 40 min post infection in PA^+Sia^ infected macrophages than PA^−Sia^ infection.

### Genetic Knockdown of Siglec-E Reveals Its Role in Modulating Macrophage Response Against PA^+Sia^

**Figure 8:**
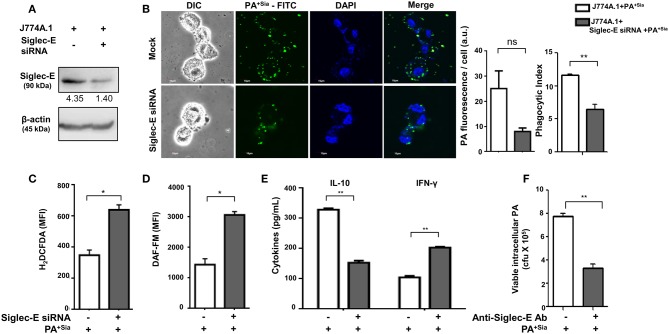
Disruption of sialic acid-siglec-E interaction by siglec-E siRNA reverses macrophage response to PA^+Sia^. **(A)** Lysates from mock and siglec-E siRNA transfectant macrophages were analyzed by western blotting to confirm silencing of siglec-E. Western blot shown is representative of three independent experiments. **(B)** Mock transfectant or siglec-E knockdown macrophages were incubated with 10 MOI FITC-PA^+Sia^ for 30 min at 37°C. Cells were visualized by confocal microscopy to check for bacterial internalization as described in Materials and Methods. Microscopy images were representative of three independent experiments. Scale bar = 10 μm. **(C)** ROS generation was estimated by CM-H2DCFDA staining in these cells and analysis by flow cytometry. **(D)** In a similar setup, macrophages were stained with DAF-FM after PA^+Sia^ infection and analyzed by flow cytometry to detect levels of reactive nitrogen species. MFI data shown **(C,D)** are as mean ± s.e.m. from three independent experiments. **(E)** Mock and siglec-E siRNA transfected cells were stimulated with PMA and infected with PA^−Sia^ or PA^+Sia^. Amount of IL-10 (TH2) and IFN-γ (TH1) cytokines secreted by macrophages in response to infection was measured by ELISA kits from culture supernatant collected after 3 h of infection. **(F)** Viability of bacteria in macrophages following phagocytosis was assessed using gentamicin protection assay. Macrophages were incubated with siglec-E blocking antibody at 4°C for 30 min followed by infection with PA^+Sia^ at 1:10 MOI for 30 min at 37°C. Infected macrophages were then incubated for 3 h with gentamicin (200 μg/mL). The viability and persistence of internalized bacteria were checked by plating the lysed macrophage contents on nutrient agar plates and checking for growing bacterial colonies (colony forming units, cfu). Data represented as mean ± s.e.m. of three independent experiments. Data from three independent experiments represented as mean ± s.e.m. Significance represented by ^ns^*p* > 0.05, *p ≤ 0.05, **p ≤ 0.01.

The role of sialic acid-siglec-E interaction on macrophage responses against PA^+Sia^ was further established by silencing expression of siglec-E in J774A.1. Reduction in siglec-E expression due to transient siRNA transfection was confirmed by western blotting (Figure 8A). Consequently, confocal microscopy revealed 3-fold less PA^+Sia^ were internalized by siglec-E knockdown macrophages (Figure 8B). Phagocytic index of PA^+Sia^ infected macrophages was 11.62 ± 0.19 while it decreased to 6.467 ± 0.72 upon siglec-E knockdown.

Subsequently, we have observed enhanced ROS generation in siglec-E silenced macrophages in response to PA^+Sia^ (MFI = 640.5 ± 31.50, *p* ≤ 0.05) as compared to mock-transfected PA^+Sia^-infected macrophages (MFI = 349.8 ± 32.20; Figure 8C). Similarly, reactive nitrogen species generation in these PA^+Sia^ -infected macrophages was higher (MFI = 2,569 ± 609.0) in absence of siglec-E rather than in its presence (MFI = 1,436 ± 195.0, *p* ≤ 0.05; Figure 8D). Siglec-E silenced PA^+Sia^-infected macrophages also showed increased IFN-γ levels (203.0 ± 3.00, *p* ≤ 0.01) and reduced IL-10 secretion (153.2 ± 6.76 pg/mL, *p* ≤ 0.01; Figure 8E). Cytokine levels of siglec-E expressing macrophages upon PA^+Sia^ infection were characterized by higher levels of IL-10 (328.7 ± 4.33 pg/mL) and lower levels of IFN-γ (105.0 ± 5.05 pg/mL). Thus, siglec-E knockdown alters macrophage cytokine response against PA^+Sia^ from a TH2 anti-inflammatory type to a pro-inflammatory TH1 type, as characterized by these two cytokines. Survival of PA^+Sia^ in siglec-E blocked macrophages was also checked by gentamicin protection assay. In absence of interacting siglec-E, the number of PA^+Sia^ that remains viable inside macrophages also decreases after 3 h of infection (7.73 ± 0.29 × 10^5^ vs. 3.31 ± 0.34 × 10^5^, *p* ≤ 0.01; Figure 8F). These observations suggest that absence of siglec-E results in stronger anti-bacterial response by macrophages leading to PA^+Sia^ elimination.

**Figure 9:**
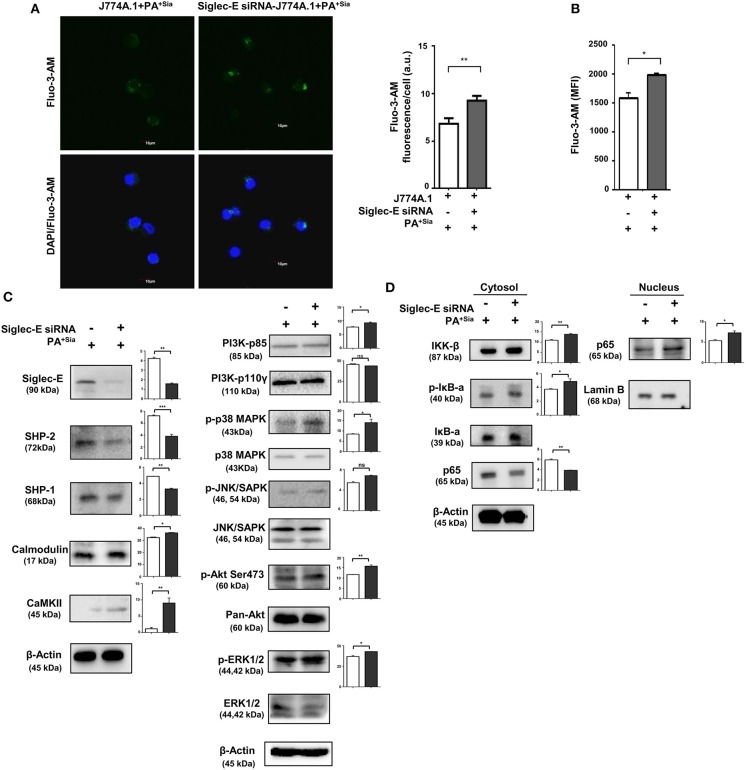
Siglec-E silenced macrophages show enhanced intracellular calcium levels, calcium-related, MAPK and NF-κB signaling in response to PA^+Sia^. **(A)** Mock and siglec-E siRNA transfected J774A.1 cells were stained with Fluo-3-AM followed by PA^+Sia^ infection for 15 min at 37°C. Cells were visualized by confocal microscopy as before. Microscopy images were representative of three independent experiments. Scale bar = 10 μm. **(B)** Intracellular calcium levels were detected by flow cytometric analysis of Fluo-3-AM stained macrophages following 15 min of 10 MOI PA^+Sia^ infection at 37°C. MFI data from three independent experiments represented as mean ± s.e.m. **(C)** Expression of MAPK, ERK, JNK pathway molecules signaling molecules and calmodulin, calmodulin-dependent kinase type II was detected in lysates from PA^+Sia^-infected mock or siglec-E siRNA transfected macrophages by western blotting. **(D)** Similarly, infected macrophages were separated into cytosolic and nuclear fractions. Proteins were resolved in SDS-PAGE and probed for the expression of NF-κB signaling pathway to detect nuclear translocation of p65 subunit. Western blots **(C,D)** were representative of three independent experiments. Densitometric values reported are normalized with respect to β-actin band intensities and such values from atleast three independent experiments are used to calculate the mean band intensities which are presented as bar diagrams alongwith statistical significance of observed changes. Significance represented by *p ≤ 0.05 and **p ≤ 0.01.

We have observed that phagosome maturation is altered in PA^+Sia^-infected macrophages (Figures 4, 5). We have also observed modulation of intracellular calcium levels upon PA^+Sia^ infection, which is known to play a significant role in such phagolysosome fusion inhibition (Figure 6). By microscopy, we identified that siglec-E has a role in such modulation, where we found higher intracellular calcium concentrations in siglec-E silenced macrophages (9.267 ± 0.29 a.u., *p* ≤ 0.01) compared to mock-transfected macrophages (6.833 ± 0.33 a.u.; Figure 9A) upon PA^+Sia^ infection. This observation was corroborated by flow cytometry, where we observed that PA^+Sia^-infected siglec-E silenced macrophages show higher calcium levels (MFI = 1,994 ± 17.50, *p* ≤ 0.05) as compared to infected mock-transfected macrophages (MFI = 1,583 ± 92.00; Figure 9B). Additionally, we also demonstrated that calcium monitoring proteins such as calmodulin and calmodulin-dependent kinase II were also elevated upon siglec-E knockdown in macrophages (Figure 9C).

We have also confirmed that siglec-E knockdown resulted in reduction in expression of SHP-2 and SHP-1 in these PA^+Sia^ infected transfected cells (Figure 9C).

Furthermore, we have observed elevated phosphoproteins of p-P38, p-AKT (Ser473), p-ERK1/2, and p-JNK/SAPK in PA^+Sia^-infected siglec-E silenced macrophages compared to mock transfectants (Figure 9C). Moreover, knockdown of siglec-E resulted in an increase in IκB-α phosphorylation and translocation of NF-κB p65 subunit from the cytosol into the nucleus of PA^+Sia^-infected macrophages (Figure 9D).

Statistical analysis of densitometry values of each band from three independent western blots were performed in all cases to evaluate the significance of observed differences in band intensities.

All these observations support that siglec-E plays an important role in regulating antimicrobial responses, intracellular calcium levels and key cellular signaling molecules in PA^+Sia^-infected macrophages.

### Infection With PA^+Sia^ Results in Altered Cellular Signaling

**Figure 10:**
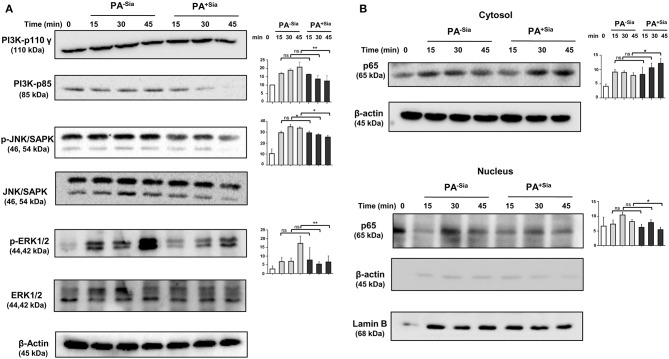
Cellular signaling pathways were altered in J774A.1 macrophages in response to PA^−Sia^/PA^+Sia^ infection. **(A)** J774A.1 were infected with PA^−Sia^ or PA^+Sia^ for 15, 30, or 45 min. A few molecules involved in cellular signaling pathways were detected by Western blotting. After visualization of phosphorylated forms of JNK, ERK, the same blots were stripped and processed to detect its total counterparts. **(B)** The expression levels of NF-κB pathway molecules and nuclear translocation of p65 subunit was assessed in cytosolic and nuclear fractions of infected macrophages. Western blots shown are representative of at least three independent experiments. Densitometric values reported are normalized with respect to β-actin band intensities. Densitometric values from at least three independent experiments were used to calculate average values of band intensity which are presented as bar diagrams with statistical significance.

So far we have demonstrated the important role of siglec-E in sialic acid-siglec-E interactions in PA^+Sia^ infected macrophages (Figure 9). Now, the status of a few key signaling molecules, previously identified to be affected by sialic acid-siglec-E interactions were also checked in PA^−Sia^/PA^+Sia^ infected macrophages at different time points post-infection (Figures 9C,D). We have observed that expression of PI3K p85 and phosphorylated forms of ERK1/2, JNK/SAPK are consistently lower in case of PA^+Sia^-infected macrophages than PA^−Sia^ infection (Figure 10A). Average of densitometric values of observed protein bands normalized with respect to β-actin bands are reported as bar diagrams and statistical significance is also reported.

We also observed that the p65 subunit of NF-κB pathway remains in the cytosol in PA^+Sia^ infected macrophages, resulting in lower activation of NF-κB pathway. However, p65 translocates into the nucleus of macrophages post infection with PA^−Sia^ (Figure 10B) indicating activation of NF-κB pathway.

## Discussion

The rapid emergence of antibiotic-resistant strains makes treatment of PA infections quite challenging, causing worldwide concerns (32, 33). Furthermore, PA possesses an arsenal of enzymes, virulence factors and a type III secretion system that play significant roles in its pathogenesis (34). Macrophages and neutrophils are innate immune cells which quickly respond to microbial infections by phagocytosing invading bacteria. In order to survive, several pathogens have devised methods to evade detection or prevent such killing by innate immune cells. Presence of sialic acid on PA has earlier been demonstrated as an important determinant for their survival against neutrophils (7). Therefore, we wanted to explore if sialic acids of PA have any impact on its interaction with macrophages.

The main finding of our current investigation includes a demonstration of higher binding and internalization of PA^+Sia^ through interactions with siglec-E present on macrophages. Such sialic acid-siglec-E interaction exhibited reduced respiratory burst and anti-inflammatory cytokine secretion. More importantly, siglec-E plays an important role in modulating calcium signaling which leads to impaired phagosome maturation thereby interrupting phagosome-lysosome fusion. Such sialic acid-siglec-E interaction also modulates several signaling molecules in MAPK and NF-κB pathways. Taken together, PA^+Sia^ utilizes host immune receptor (siglecs) strategically to impair macrophage immune response leading to their survival and persistence inside the macrophage.

In case of PA infections in lower airways, alveolar macrophages uptake these bacteria mostly by non-opsonic phagocytosis through complement receptor-3 and CD14 receptors for their elimination from the lung (12, 35). We have observed similar binding of PA^+Sia^ to macrophages through complement receptor-3 (Supplementary Figure S1). Most importantly, we have identified that PA additionally utilizes sialic acid for higher binding and increased internalization inside both human and murine macrophages. Desialylation of PA^+Sia^ reduced this enhanced binding indicating a critical role of sialic acids (ligands) in such binding.

Immune receptor siglecs mediate binding to sialic acids. Siglec-F, siglec-G and siglec-H are present on mouse mast cells, B cells, and plasmacytoid dendritic cells, respectively (36). Murine macrophages express mostly siglec-1 and siglec-E. Siglec-E is involved via sialic acids by several pathogens leading to modulation of innate immune response (36–40). Here we have demonstrated that siglec-E on J774A.1 and siglec-9 on THP-1 are two important immune receptors responsible for mediating higher binding and internalization of PA^+Sia^ (Figure 2).

Macrophages eliminate phagocytosed bacteria by chemokine and cytokine secretion, phagosomal acidification, ROS generation and finally phagolysosome formation (9). Oxygen and nitrogen radical generation by macrophages effectively eliminates PA in cystic fibrosis patients as well (41). TH1 type cytokine responses lead to better clearance of PA in the lungs and higher survival rates of infected mice (42). Here we report that antimicrobial responses such as ROS and RNS generation were suppressed in PA^+Sia^-infected macrophages. This corroborates our previous report that PA^+Sia^ interactions with siglec-9 on neutrophils also result in a similar reduction in respiratory burst (7). Also, PA^+Sia^-infected macrophages show an anti-inflammatory cytokine response, characterized by higher levels of IL-4, IL-12, and TGF-β secretion. Thus, macrophage response against PA^+Sia^ is impaired. However, interruption of sialic acid-siglec interactions by desialylation of PA^+Sia^ or blocking or silencing siglec-E on macrophages results in reversal of such innate immune responses.

Following internalization, bacteria bound in phagosomes may undergo fusion with lysosomes. A lysosome is a highly acidic organelle containing enzymes like lysozymes, proteases, lipases and cathepsins along with redox stressors responsible for breakdown of invading microbes, exogenous particles, apoptotic bodies or damaged cellular organelles (9, 43). We followed the trafficking of PA^+Sia^ phagosomes through the different stages of phagosome maturation using both microscopy as well as flow cytometry. At initial time points, higher numbers of PA^+Sia^ were localized in early endosomes (Rab5 positive), consistent with our observations that more number of PA^+Sia^ were internalized. However, fewer bacteria were found in the late phagosomal compartment (Rab7 positive) at both 20 and 40 min post infection (Figures 5B,E). Since Rab7 mediates the final fusion with lysosomes (labeled by LAMP1), inhibition of Rab7 recruitment may have caused lowered phagosome maturation in PA^+Sia^-infected macrophages (Figure 4). Taken together, lowered respiratory burst, anti-inflammatory cytokines and inhibition of phagosome-lysosome fusion lead to impaired killing of PA^+Sia^. This was confirmed by gentamicin protection assay where PA^+Sia^ remained viable following 3 h after internalization inside the macrophages. Interestingly, we have observed that PA^+Sia^ also undergo cellular division inside the macrophages through dilution of CFSE dye (Figure 4).

Current research has raised questions about the generalization that PA is only an extracellular pathogen as it has also been found to exist intracellularly (44–46). The toxin ExoS has been implicated in inhibiting phagosome-lysosome fusion in infected epithelial cells, allowing PA to survive intracellularly in membrane blebs (46–48). ExoS also interacts with Rab5 thereby preventing phagocytosis of bacteria (49). A study involving human and mouse primary macrophages has also reported that PA is capable of entering, surviving as well as multiplying intracellularly in macrophages, depending on expression of bacterial outer membrane protein—OprF (50). Other factors from PA such as mgtC and oprF allow PA to persist intracellularly in macrophages (45, 51). There are several indications that PA can influence endocytic pathways for their intracellular existence. Here, we have observed that macrophages fail to completely eliminate PA^−Sia^ or PA^+Sia^. However, we have demonstrated that PA^+Sia^ utilizes bacterial surface sialic acids to interact with siglecs on macrophage surface as a strategy to enhance its internalization as well as intracellular survival.

The phenomenon of phagosome-lysosome fusion inhibition has been extensively studied in a few bacteria like *Mycobacterium* sp., *Salmonella* enteric serovar *typhimurium*, and *Francisella tularensis* (52). Decrease in intracellular calcium concentrations and altered calcium signaling results in inhibition of phagosome-lysosome fusion in *Mycobacterium* infection (30). However, the role of intracellular calcium in modulating phagosome-lysosome fusion is controversial (30, 31, 53–58). In an *in vitro* system, calcium has been demonstrated to be essential for fusion between late endosomes and lysosomes (59). Furthermore, enhanced intracellular calcium also induces phagosome-lysosome fusion and inhibits bacterial viability (30). In murine macrophages interacting with unopsonized yeast cells, phagosome maturation is reported to be regulated by intracellular calcium concentrations (58). Particularly, phagocytosis of PA also depends on the increase in intracellular calcium concentration mediated by TRPV2- a calcium channel on macrophage plasma membrane (60). Here, we also demonstrate that a reduction in intracellular calcium concentration accompanied by lower expression of calcium-sensing molecules is correlated with the inhibition of phagosome maturation during PA^+Sia^ infection (Figures 4, 6). Also, ionomycin-mediated increase in intracellular calcium actually enhanced phagosome-lysosome fusion rate in PA^+Sia^-infected macrophages (Figure 6) indicating that intracellular calcium concentration modulates phago-lysosome fusion in PA^+Sia^-infected macrophages.

Increasing the extracellular Ca^2+^ concentration also lead to reduction of viability of internalized PA^−Sia^/PA^+Sia^ but the mechanism of bacterial clearance was not due to phagosome-lysosome fusion. Interestingly, extracellular calcium abrogates the anti-inflammatory response observed in PA^+Sia^-infected macrophages. Together with the reduction in viability of internalized PA^+Sia^, this might suggest that extracellular calcium, at higher concentrations, may improve anti-bacterial response by macrophages.

Certain calcium-sensitive proteins like Calmodulin and calmodulin type kinase II play an important role in phagolysosome fusion in *Mycobacterium* infected macrophages. Calmodulin is known to be involved in phagosome maturation and phagolysosome formation both in cell-free systems and bacteria-infected cells (30, 61–64).

Calmodulin also plays a role in antimicrobial responses by associating with inducible nitric oxide enzyme (iNOS) and enhancing nitric oxide production in macrophages (65). In the case of PA^+Sia^-infected macrophages, lowered calmodulin levels, and reduced nitric oxide production hints at this relation. In a calcium-dependent manner, calmodulin activates calcineurin, which interacts with a group of transcription factors like the calcineurin-dependent nuclear factor of activated T cells (NFAT). Calcineurin-NFAT signaling is also known to collaborate with NF-κB signaling and induces pro-inflammatory cytokine production (66). Here we report that calcineurin expression was lowered in PA^+Sia^-infected macrophages from 45 min post infection, which may impact such calcineurin-dependent signaling events leading to an anti-inflammatory cytokine response.

Thus, calcium signaling and related pathways are modulated due to sialic acid-siglec-E interactions. Reduced intracellular calcium levels as well as calcium-sensing proteins calmodulin and calmodulin kinase II were reversed by silencing siglec-E suggesting an important contribution by siglec-E in modulating calcium in PA^+Sia^-infected macrophages.

These results indicate that siglec-E can possibly function like siglec-G (CD22) on B cells which inhibits calcium flux from B-cell receptor (BCR) ligation through recruitment of SHP-1 phosphatases (67). The involvement of siglec-8 in mast cells similarly inhibits calcium flux generated due to FcεRI cross-linking (68). Rise in intracellular calcium ions in macrophages during PA infection may be due to TRPV channel recruitment (60) or PA^−Sia^ binding with other receptors (69–71) which is consequently diminished in PA^+Sia^-infected macrophages. We have demonstrated an increased phosphorylation of tyrosine residues of siglec-E and enhanced association of siglec-E and phosphatases SHP-1/SHP-2 in PA^+Sia^-infected macrophages. In a similar fashion, siglec-E mediated SHP-1/2 recruitment in case of PA^+Sia^ infection may be responsible for lowered intracellular calcium levels in contrast to PA^−Sia^. Taken together, we may propose that this sialic acid-siglec-E interaction modulate macrophage intracellular calcium levels thereby decreasing phagosome-lysosome fusion in PA^+Sia^-infected macrophages.

A contrasting report by Perdicchio et al. indicated that sialylation does not affect the intracellular routing of antigens hence artificially sialylated ovalbumin reached lysosomes like unmodified ovalbumin (72). However, virulence factors of PA are known to modify host cellular responses, including modulating the endocytic pathways (51). Interactions between such a sialylated bacterial pathogen with macrophages are fundamentally different from that involving artificially sialylated antigen uptake by bone marrow derived dendritic cells (BM-DCs).

The impact of siglec-E-sialic acid interactions on cellular signaling was convincingly demonstrated by silencing siglec-E which reduced PA^+Sia^ association with macrophages but restored the antimicrobial response via high ROS, RNS generation and pro-inflammatory cytokine secretion. Additionally, MAPK pathway and NF-κB translocation were also modulated by such silencing.

**Figure 11:**
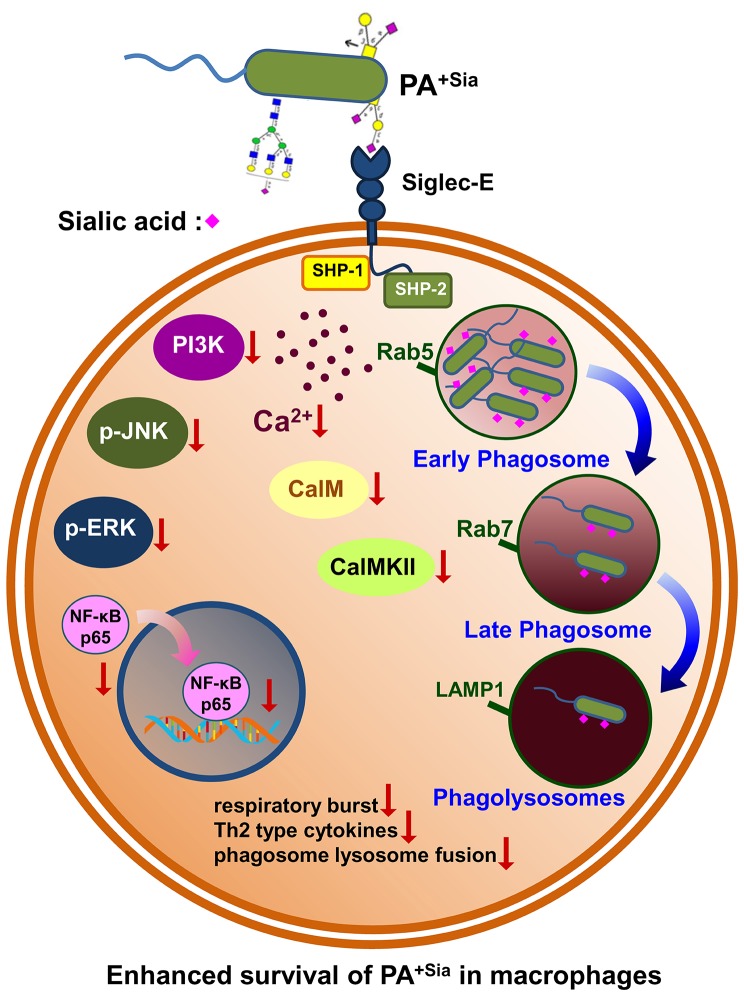
PA^+Sia^ modulates macrophage responses to promote its survival inside macrophages through sialic acid-siglec-E involvement. The schematic diagram summarizes the altered macrophage immune response against PA^+Sia^ due to involvement of siglec-E which leads to persistence of PA as an intracellular pathogen.

Taken together, we have demonstrated that PA^+Sia^ are internalized more by both murine and human macrophages. Such sialic acid-siglec interactions influence anti-bacterial responses like respiratory burst, modulation of intracellular calcium flux and calcium related signaling molecules. All these events lead to altered cytokine response through MAPK, ERK and NF-κB signaling pathways and ultimately inhibition of phagosome-lysosome fusion. As a result, the phagocytosed PA^+Sia^ are comparatively eliminated less and found to persist intracellularly in the macrophages establishing the critical role of sialic acids and siglec-E for bacterial persistence. Our findings regarding the role of sialic acid-siglec-E interactions in PA pathogenesis of macrophages is illustrated in a schematic diagram (Figure 11).

Therefore, the potential payback of our work is a greater understanding of the roles of sialoglycans and the function of siglec-E in interactions between sialylated bacteria and immune cells as a way to better understand bacterial pathogenesis.

## Data Availability Statement

All datasets generated for this study are included in the article/Supplementary Material.

## Author Contributions

BK conceived the work and initiated the project. KM performed most of the experiments and wrote the manuscript. CM supervised the whole work, evaluated, and corrected the manuscript. All authors have contributed valuable comments and scientific inputs to the manuscript writing.

### Conflict of Interest

The authors declare that the research was conducted in the absence of any commercial or financial relationships that could be construed as a potential conflict of interest.
