# Supplementary figures and images for: Sialic Acid-Siglec-E Interactions During Pseudomonas aeruginosa Infection of Macrophages Interferes With Phagosome Maturation by Altering Intracellular Calcium Concentrations
Source: Front Immunol. 2020 Feb 28;11:332. doi: 10.3389/fimmu.2020.00332 (PMC7059019; doi:10.3389/fimmu.2020.00332)

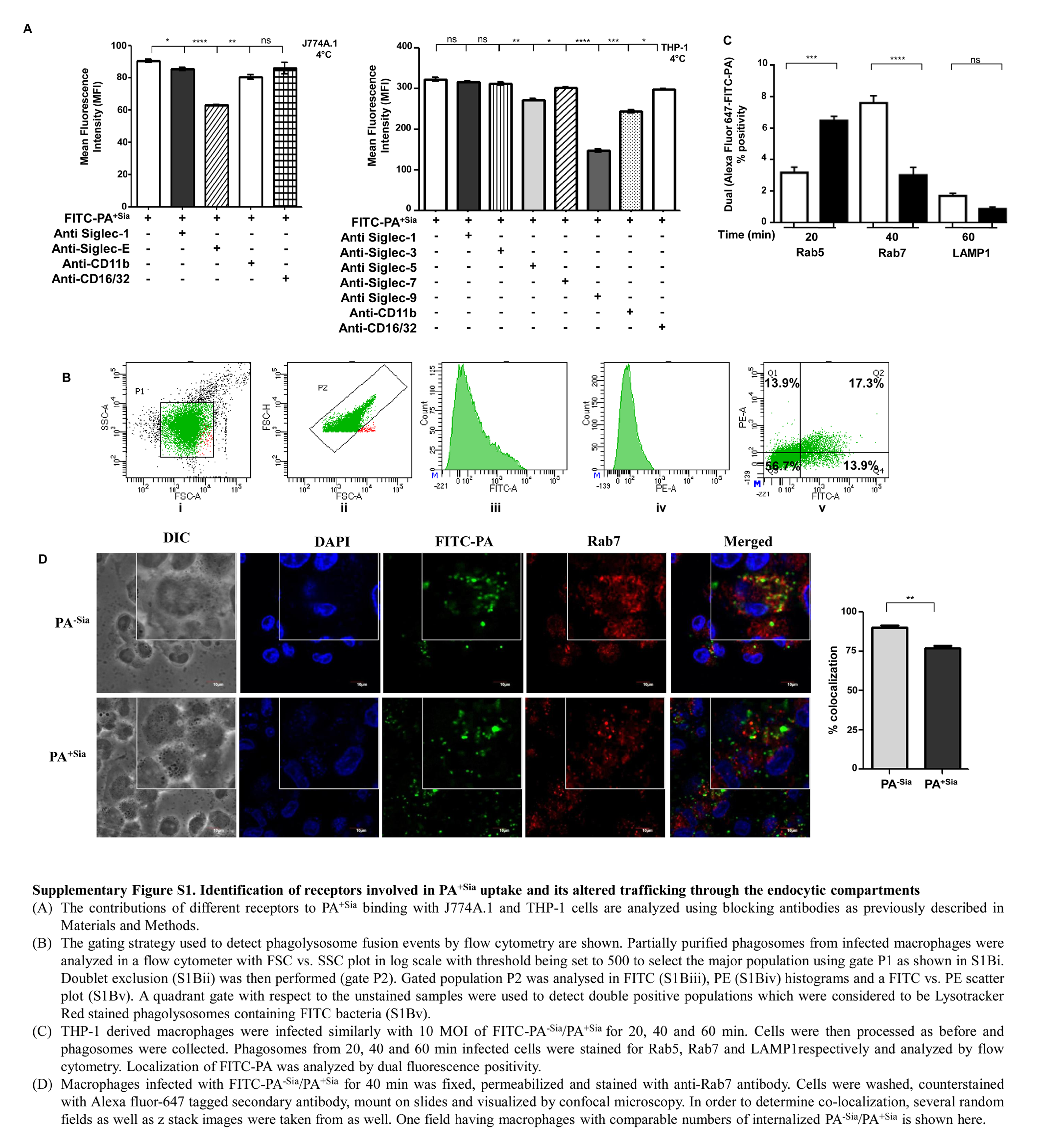

Supplement: Supplementary file 1 [file Image_1.TIF]
